# CircSMARCC1 facilitates tumor progression by disrupting the crosstalk between prostate cancer cells and tumor-associated macrophages via miR-1322/CCL20/CCR6 signaling

**DOI:** 10.1186/s12943-022-01630-9

**Published:** 2022-09-01

**Authors:** Tao Xie, Du-jiang Fu, Zhi-min Li, Dao-jun Lv, Xian-Lu Song, Yu-zhong Yu, Chong Wang, Kang-jin Li, Baoqian Zhai, Jiacheng Wu, Ning-Han Feng, Shan-Chao Zhao

**Affiliations:** 1 Department of Urology, Nanfang Hospital, Southern Medical University, Guangzhou, 510515 China; 2 Department of Urology, the Third Affiliated Hospital of Southern Medical University, Guangzhou, 510500 China; 3 Department of Urology, the Third Affiliated Hospital of Guangzhou Medical University, Guangzhou, 510150 China; 4 Department of Radiotherapy, Affiliated Cancer Hospital & Institute of Guangzhou Medical University, Guangzhou, 510095 China; 5 Department of Radiotherapy Oncology, Yancheng City No.1 People’s Hospital, Yancheng, 224005 China; 6 The Fourth Affiliated Hospital of Nantong University, Yancheng, 224005 China; 7 Department of Urology, Affiliated Tumor Hospital of Nantong University & Nantong Tumor Hospital, No. 30 Tongyang bei Road, Tongzhou District, Nantong, 226361 China; 8 Department of Urology, Affiliated Wuxi No. 2 Hospital, Nanjing Medical University, Wuxi, 214002 China

**Keywords:** Prostate cancer, Epithelial mesenchymal transformation, CircSMARCC1, CC-chemokine ligand 20, Tumor-associated macrophage

## Abstract

**Background:**

Circular RNAs (circRNAs) mediate the infiltration of tumor-associated macrophages (TAMs) to facilitate carcinogenesis and development of various types of cancers. However, the role of circRNAs in regulating macrophages in prostate cancer (PCa) remains uncertain.

**Methods:**

Differentially expressed circRNAs in PCa were identified by RNA sequencing. The expression of circSMARCC1 was recognized and evaluated using fluorescence in situ hybridization and quantitative real-time PCR. The oncogenic role of circSMARCC1 in PCa tumor proliferation and metastasis was investigated through a series of in vitro and in vivo assays. Finally, Western blot, biotin-labeled RNA pulldown, luciferase assay, rescue experiments, and co-culture experiments with TAMs were conducted to reveal the mechanistic role of circSMARCC1.

**Results:**

CircSMARCC1 was dramatically up-regulated in PCa cells, plasma and tissues. Overexpression of circSMARCC1 promotes tumor proliferation and metastasis both in vitro and in vivo, whereas knockdown of circSMARCC1 exerts the opposite effects. Mechanistically, circSMARCC1 regulates the expression of CC-chemokine ligand 20 (CCL20) via sponging miR-1322 and activate PI3K-Akt signaling pathway involved in the proliferation and epithelial mesenchymal transformation. More importantly, high expression of circSMARCC1 was positively associated with colonization of CD68^+^/CD163^+^/CD206^+^ TAMs in tumor microenvironment. In addition, overexpression of circSMARCC1 facilitates the expression of CD163 in macrophages through the CCL20-CCR6 axis, induces TAMs infiltration and M2 polarization, thereby leading to PCa progression.

**Conclusions:**

CircSMARCC1 up-regulates the chemokine CCL20 secretion by sponging miR-1322, which is involved in the crosstalk between tumor cells and TAMs by targeting CCL20/CCR6 signaling to promote progression of PCa.

**Supplementary Information:**

The online version contains supplementary material available at 10.1186/s12943-022-01630-9.

## Background

Prostate cancer (PCa) is the second most frequent cancer and the fifth-leading cause of cancer-related death in men [1]. Many therapeutic strategies have offered the opportunity for the cure of PCa, including radical prostatectomy or radiation therapy. Unfortunately, most PCa patients are often diagnosed in an advanced stage in which radical prostatectomy cannot be performed leading to poor prognosis. The 5-year relative survival rate for localized PCa is close to 100%, compared to 30% for advanced metastatic prostate cancer (mPCa) [2]. Therefore, exploring tumor-related biomarkers and understanding the molecular events of PCa will be useful for early diagnosis and efficient treatments.

Circular RNAs (circRNAs) are a group of endogenous non-coding RNA molecules that are linked head to tail by reverse splicing to form a covalently closed loop structure [3]. Growing number of studies have revealed that circRNAs play critical roles in regulating tumor proliferation and metastasis of multiple human malignancies [4]. They may act as miRNA decoys [5], RNA-binding protein sponges and protein scaffolds [6], transcriptional regulators [7], or templates for protein translations [8]. Due to their high stability, broad expression and tissue specificity, circRNAs are emerging as promising biomarkers and therapeutic targets for cancers. Several circRNAs, such as circSMARCA5 [9], hsa_circ_0003258 [10] and circPFKP [11] have been found to impact the development and progression of PCa. However, the specific roles and mechanism of circRNAs in PCa progression are largely unknown.

The tumor microenvironment (TME) is a highly heterogeneous ecosystem that typically contains a collection of tumor cell populations, immune cells, and tissue-specific resident and recruited stromal cell types [12]. Tumor-associated macrophages (TAMs) are an important component of the TME and displays two major phenotypes, M1 and M2. It is known that M1 macrophages can be polarized by lipopolysaccharide (LPS) and interferon-γ (IFN-γ), whereas M2 macrophages are polarized in the presence of IL-4 and IL-13 [13, 14]. Increased TAMs infiltration is associated with advanced disease and poor overall survival in breast cancer [15], pancreatic cancer [16] and bladder cancer [17]. Therefore, targeting TAMs infiltration could be a promising target for cancer therapy.

Here, we conducted circRNA sequencing in the plasma of PCa patients to identify circRNAs that are involved in PCa progression. For the first time, we reported a novel circRNA, named circSMARCC1 (circBase ID: hsa_circ_0001296), which is highly expressed in PCa tissue samples and cell lines. Then, gain- and loss-of-function experiments were conducted to reveal the biological roles of circSMARCC1 in cell growth, invasion and metastasis both in vitro and in vivo. Mechanistically, we found that circSMARCC1 increases CC-chemokine ligand 20 (CCL20) by sponging miR-1322 and activates the PI3K-Akt pathway to promote growth and epithelial mesenchymal transformation (EMT) of PCa cells. More importantly, we found that circSMARCC1 promotes recruitment of macrophage and M2 polarization, which in turn facilitates the progression of PCa. Our findings indicate that circSMARCC1 could be a promising therapeutic target for PCa.

## Materials and methods

### Patients and clinical samples

The plasma of PCa patients and BPH individuals used in this study were obtained from patients who were hospitalized at Nanfang Hospital of Southern Medical University (Guangzhou, China). Formalin-fixed paraffin-embedded PCa specimens were acquired from patients that underwent radical prostatectomy at Nanfang hospital, and patients’ clinical information was obtained by reviewing the follow-ups of their electronic medical records. All experimental procedures were approved by the Medical Ethics Committee of Nanfang Hospital of Southern Medical University (NFEC-2022-083). The selection criteria for PCa patients are as follows. Inclusion criteria: 1) Pathologically confirmed prostate adenocarcinoma; 2) New cases without any preoperative treatment; 3) Patients signed informed consent. Exclusion criteria: 1) The pathological examination type was neuroendocrine prostate cancer (NEPC)/small cell prostate cancer; 2) Combined with other tumors; 3) Patients with other acquired, congenital immunodeficiency disease, severe liver, kidney or other systemic diseases, or a history of organ transplantation; 4) Patients who received preoperative chemotherapy or radiotherapy before surgery. The median age of the enrolled patients was 65.5 years, and the average age was 66 (range: 48–81 years). Clinical TNM staging and Gleason scores of patients were based on the American Joint Committee on Cancer Eighth Edition (2017) and the 2016 World Health Organization classification of genitourinary tumors.

### Cell culture and treatment

The human monocyte THP-1cells, human embryonic kidney HEK-293 T cells, normal prostate epithelial RWPE-1 cells and PCa cells (PC-3, DU145, 22Rv1, C4–2 and LNCaP) were obtained from the Cell Bank of the Chinese Academy of Sciences. Cells were grown in RPMI-1640 medium (Gibco, United States) supplemented with 10% FBS (Gibco, United States). All cells were maintained at 37 °C with 5% CO_2_. Three small interfering RNAs (siRNAs) targeting circSMARCC1 (si-circ # 01, 02, 03) and miR-1322 inhibitors or mimics were purchased from RiboBio Company (Guangzhou, China). Cell lines stably overexpressed circSMARCC1(lv- circSMARCC1) or knocked down circSMARCC1(sh-circSMARCC1) were established using lentivirus vectors (GeneChem Bio-Medical Biotechnology, Shanghai, China), and the transfected cells were selected in puromycin (2 g/ml) for 1 week. All the target sequences are shown in Supplementary Table S1–2.

### CircRNA microarrays

The circRNAs from the plasma of PCa patients and control individuals for microarray analysis were based on the previous protocols [18]. Arraystar Human circRNA Array v2 (Kangcheng Biotech, Shanghai, China) was applied to the analysis of the circRNA microarray. Sample preparation was performed according to the Arraystar standard protocols, as described previously [19]. Differentially expressed circRNAs were identified via fold change filtering. We defined the statistical criteria for selecting differentially expressed circRNAs using fold change > 1.5 with *p* < 0.05.

### RNA extraction, nuclear-cytoplasmic fractionation, RNase R and actinomycin D treatment, and qRT-PCR assays

Total RNA was extracted from tissues or cell lines using TRIzol reagent (Takara, Dalian, China). RNAs from the nucleus and cytoplasm of PCa cells were separated by Nuclear/Cytoplasmic Isolation Reagent (Thermo Fisher Scientific, United States) following the manufacturer’s instructions. Quantitative real-time PCR (qRT-PCR) was applied for further detection. For RNase R treatment, cells were treated with 2 mg of total RNA for 10, 20, and 30 min at 37 °C with 3 U/g of RNase R (Epicentre Biotechnologies, Madison, WI, United States). In addition, total RNA from PCa cells was treated with 1 μg/ml actinomycin D (Cell Signaling Technology, Beverly, MA, United States) against new RNA synthesis for 0, 4, 8, 12, and 24 h. RNA was reversely transcribed into cDNAs with the PrimeScript RT reagent Kit (Takara, Dalian, China) according to the manufacturer’s instructions. SYBR Green PCR Master Mix (Takara, Dalian, China) and the Applied Bio-systems 7500 Fast Real-Time RCR System (Applied Biosystems, United States) were used for RT-qPCR analysis. Each measurement was performed in triplicate and the results were standardized against the internal control GAPDH. The relative expression of target genes was calculated using the 2^**-△△Ct**^ method. All the primers used are shown in Supplementary Table S3.

### Fluorescence in situ hybridization (FISH)

Cy3-labeled circSMARCC1 (RiboBio, Guangzhou, China) and Alexa 488-labeled miR-1322 probes (FOCOFISH, Guangzhou, China) were used to observe the co-localization of circSMARCC1 and miR-1322 in PCa tissues and cells. The FISH experiment was conducted using a Fluorescent in Situ Hybridization Kit (No. C10910, RiboBio, Guangzhou, China), according to the official guidelines. Cell nuclei were stained with 4,6-diamidino-2-phenylindole (DAPI, Beyotime, China). The images were photographed under the fluorescence microscope (LSM 880 with Airyscan, Carl Zeiss, Germany).

### Cell proliferation, scratch assay, migration and invasion assays

CCK-8, EDU, colony formation, scratch assay, transwell migration, and invasion assays were performed as previously reported [20]. Specifically, for cell proliferation assays, stably transfected PCa cells were treated with the conditioned medium (CM) from TAMs (co-cultured with PCa cells), human recombinant CCL20 protein (rh-CCL20, 20 ng/ml, #0511102, Peprotech) and neutralizing antibody to CCL20 (anti-CCL20, 5 μg/ml; ab9829, Abcam). The cell proliferative rate was assessed using the Cell Counting Kit-8 (CK-04, Dojindo). For tumor cell migration assays, the stable cells lines treated with or without 5 μg/ml CCL20 neutralizing antibody were seeded in the upper chambers; the lower chambers were filled with medium containing 10% FBS with or without 20 ng/ml CCL20 recombinant protein. In addition, the CM of TAMs was placed in the lower chamber and used as an attractor for PCa cell migration and invasion experiments.

### Macrophage generation, macrophage migration, and co-culture assay

We induced M2 macrophage generation by sequential stimulation as follows: THP-1 cells were treated with 100 ng/ml phorbol-12-myristate-13-acetate (PMA) (Beyotime, Shanghai, China) for 24 h for differentiation into adhered THP-1 macrophages (THP-1-Mø). Then, THP-1-Mø were incubated with 20 ng/ml IL-4 (AF-200-04, PeproTech) and 20 ng/ml IL-13 (AF-200-13, PeproTech) for 48 h to obtain M2 polarization (THP-1-M2). For macrophage migration, the migration assays were performed using 6.5 mm transwell plates with 5.0 μm pore size inserts. The CM of stably transfected PCa cells with or without CCL20 recombinant protein was served as an elicitor in the lower chamber of the 24-well plate, and THP-1-M2 were added to the upper transwell insert (#09717050, Corning). To explore the potential mechanisms by which CCL20 acts, TAMs were incubated with CCR6-neutralizing antibody (anti-CCR6, #MHH-160-F(E), Creative Biolabs) before migration assays were performed. After 48 h incubation, the cells adhering to the lower filter surface were fixed with 4% paraformaldehyde for 10 min and stained with Giemsa (Baso Diagnostics, Inc., Zhu Hai, China) for transwell migration assays. For co-culture experiments, PCa cells with stably overexpressed or knocked down circSMARCC1 were inoculated into the upper insert and then transferred to 6-well plates pre-inoculated with THP-1-Mø. After 48 h, macrophages were collected for the experiments.

### Cell-cycle analysis and flow cytometry

For cell cycle analysis, human PCa cells were digested using 0.25% Trypsin/EDTA solution and fixed with ice-cold 70% ethanol at 4 °C overnight, then stained with propidium iodide (PI) (keygentec, Nanjing, China) and measured by flow cytometry (FACS Calibur, Becton Dickinson). The THP-1 macrophages were collected, washed, and incubated for 30 min at 4 °C with florescence-conjugated antibodies. To facilitate intracellular staining, cells were fixed and permeabilized with a fixation/permeabilization solution kit (BD Cytofix/Cytoperm) and 1% paraformaldehyde (PFA). For evaluating the M2 polarization of macrophages, PE-Cyanine7 Monoclonal anti-human CD68 (eBioscienc, Invitrogen) and PE Monoclonal anti-human CD163 (eBioscienc, Invitrogen) were used. The results were analyzed using the FlowJo 10.7 software program. All assays were repeated three times.

### Luciferase reporter assay

The sequences of circSMARCC1 or CCL20 3′-untranslated region containing the wild-type (Wt) or mutant (Mut) binding site of hsa-miR-1322 were designed and packaged into pEZX-MT06 vector (GeneCopoeia, Guangzhou, China). HEK-293 T cells were co-transfected with the corresponding plasmids and miR-1322 mimics/ miR-nc or miR-1322 inhibitors/inh-nc with Lipofectamine 3000 (Invitrogen, United States). The relative luciferase activity was measured utilizing Luc-PairTM Duo-Luciferase HS Assay Kit (GeneCopoeia, China). Each group was confirmed in triplicate.

### RNA pulldown assay

To pull down the miRNA by circRNA, biotinylated-circSMARCC1 probe (5′-CATCTTCCTCATCACAGCAC-3′) was synthesized by RiboBio (Guangzhou, China), and the oligo probe (5′-TATGTTGTTGATTTGCTGGC-3′) was used as a control. CircRNA pulldown assay was carried out using PierceTM Magnetic RNA-Protein Pull-Down Kit (No: 20164, Thermo Fisher Scientific, United States). All procedures followed the manufacturer’s instructions. Then the final RNA was extracted by TRIzol (Invitrogen, United States) and analyzed by RT qPCR.

### Western blot analysis

The RIPA lysis buffer containing protease inhibitors (# KGP250, KeyGEN BioTECH, Nanjing, China) was used to extract PCa cell protein following the standard protocol. Then, equal amounts of proteins in the cell lysates were separated by SDS/PAGE gels (4–12%, Bio-Rad) and electronically transferred onto polyvinylidene fluoride (PVDF, Millipore) membranes. The membranes were then blocked with 5% bovine serum albumin (BSA) and incubated overnight at 4 °C with the following specific primary antibodies: rabbit CCL20 antibody (#ab9829, Abcam) and rabbit CCR6 antibody (#ab110641, Abcam); EMT Antibody Sampler Kit (#9782), rabbit CD68(#97778) and CD163(#93498), rabbit CDK2 antibody (#18048), rabbit P27 Kip1 antibody (#3686), rabbit p21 Waf1/Cip1 antibody (#2947), rabbit Akt antibody (#4691), rabbit phospho-AktSer473 antibody (#4060), and rabbit phospho-AktThr308 antibody (#13038) were purchased from Cell Signaling Technology except for mouse β-actin antibody (#60008–1-Ig, Proteintech Group). Subsequently, horseradish peroxidase (HRP)-conjugated secondary antibody was used to incubate the samples for 1 h at room temperature. The bands were visualized using the enhanced chemiluminescence (ECL) detection system (Pierce Biotechnology, Rockford, IL, United States).

### Immunohistochemistry (IHC) and immunofluorescence (IF)

Assays were performed as previously reported [20]. In brief, for the IF experiment, cells were incubated with primary antibodies against CCL20 (1:200, #ab9829) (Abcam) at 4 °C overnight, then incubated with the fluorescent secondary antibody Alexa Fluor 594-conjugated goat anti-mouse IgG (Cell Signaling Technology) and imaged using a fluorescence microscope (DM5000B, Leica). For IHC, the paraffin sections were incubated with antibodies against CCL20 (1:200, #ab9829) (Abcam). Intensity scores were recorded as: 0 (no staining), 1 (weakly staining, light yellow), 2 (moderately staining, yellowish brown), and 3 (strongly staining, brown). In addition, we also detected the expression of ki67 (1:1000, #9449), CD68 (1:200, #97778), CD163 (1:400, #93498), CD206 (1:200, #24595) and CD31 (1:100, #77699) (Cell Signaling Technology) in PCa tissue or xenograft tissue. Images were observed under an Olympus multifunction microscope (Olympus BX51, Tokyo, Japan). All evaluations were performed by three independent senior pathologists using the same microscope.

### Enzyme-linked immunosorbent assay (ELISA)

The concentration of CCL20 was detected with a commercial ELISA kit (Human MIP-3 alpha ELISA, RayBiotech) according to the manufacturer’s instructions. The concentration of cytokines in serum or cell lysates was quantitated by comparison of the ELISA data with a standard curve obtained with known concentrations of cytokines.

### RNA-seq processing

RNA sequencing and sequence quality control of the DU145-vector and DU145-lv-circSMARCC1 cells were performed using the BGISEQ platform. The human genome reference was established from UCSC version GRCh38/hg38 chromosomes 1–22, X, Y, and mitochondrial DNA. Additional analysis, including a heatmap, gene set enrichment analysis (GSEA) was completed using the BGI Dr. Tom system. The Kyoto Encyclopedia of Genes [KEGG] was used for gene annotation of sequencing data.

### Animal models

Xenograft models were created through injection of 5 × 10^6^ DU145-vector, DU145-lv-circSMARCC1 cells (*n* = 7 per group), on the axillae of BALB/c male mice (4–5 weeks). The mice were obtained from the Animal Center of Southern Medical University, Guangzhou, China. All experimental animal procedures were authorized by the Nanfang Hospital Animal Ethics Committee of Southern Medical University (NFYY-2020-0132). The mice were raised under Specific Pathogen Free (SPF) conditions. Tumor size was measured every 5 days and the volume was calculated using the formula: volume = (length × width^2^)/2. To assess metastasis, 5 × 10^6^ cells in 100 μL of PBS were injected via the tail veins of nude mice (*n* = 7 per group). After 7 weeks, the mice were anesthetized, and D-luciferin (#D-Luciferin, Apexbio) was injected intraperitoneally. The IVIS imaging system (Caliper Life Sciences) was used to visualize the luciferase signal. IHC and hematoxylin-eosin (H&E) staining were used to evaluate the characteristics of xenograft tumors and lung metastasis.

### Statistical analysis

Data were analyzed using the student’s t test or non-parametric Mann–Whitney U Test and one-way analysis of variance (ANOVA) in GraphPad Prism version 8. The clinicopathological parameters in PCa cases were analyzed using Pearson’s chi-square test or Fisher’s exact chi-square test. The receiver operating characteristic (ROC) curve and Kaplan–Meier survival analyses were used to estimate the diagnostic and prognostic value. *P* < 0.05 was considered statistically significant. Data were presented as the means ± standard error of mean.

## Results

### Identification and characterization of circSMARCC1 in PCa

**Fig. 1:**
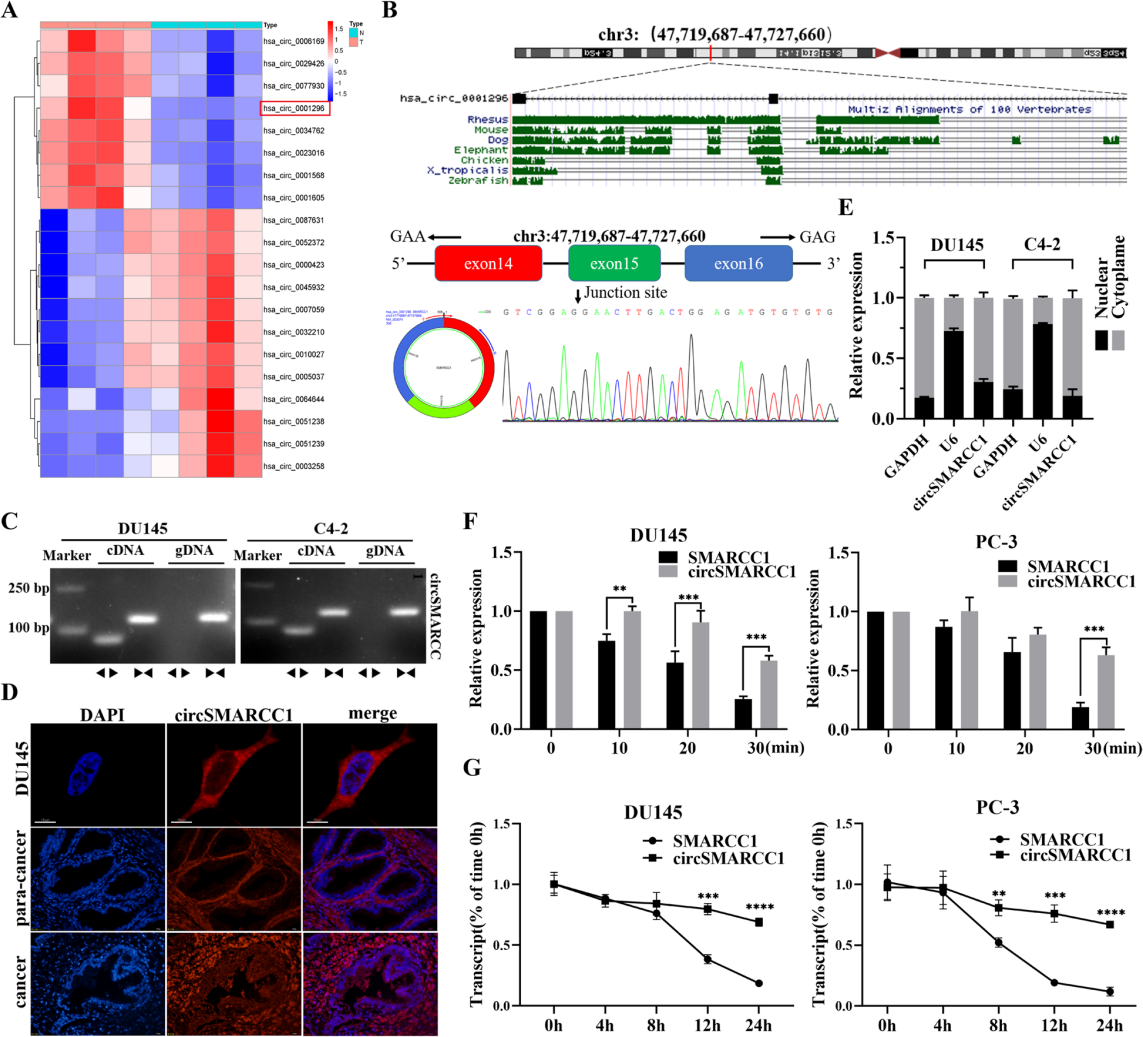
circSMARCC1 validated and characterized in PCa cells. **A** The cluster heat map demonstrated the top 20 circRNAs differentially expressed in four pairs of plasma samples from PCa and BPH patients. **B**, **C** Schematic representation of the formation of circSMARCC1 by cyclization of exons 14, 15, and 16 of the SMARCC1 gene. The back splice junction sequence and RT-PCR product of circSMARCC1 were verified by Sanger sequencing and agarose gel electrophoresis, respectively. **D** The localization of circSMARCC1 observed in PCa tissues (scale bar, 20 μm) and cells (scale bar, 10 μm) detected by FISH. Nuclei were stained with DAPI. **E** Analysis of the cellular localization of circSMARCC1 by nuclear-cytoplasmic fractionation experiment. GAPDH was used as a control for cytoplasmic proteins and U6 was used as a nuclear control. **F** qRT-PCR was performed to determine the abundance of circSMARCC1 and SMARCC1 mRNA in PCa cells treated with RNase R at the indicated time points. **G** RNA expression of circSMARCC1 and SMARCC1 in PCa cells analyzed by qRT-PCR after treatment with actinomycin D for 4 h,8 h,12 h and 24 h.The data are presented as mean ± SD.**p < 0.01, ***p < 0.001, ****p < 0.0001

Arraystar Human CircRNA Array v2 (Kangcheng Biotech, Shanghai, China) was applied to analyze the circRNA microarray [18]. To explore the circRNA expression profile in PCa, we performed RNA sequencing analyses of ribosomal RNA-depleted total RNA from 4 pairs of plasma samples of patients with benign prostatic hyperplasia (BPH) or PCa. The cluster heat map revealed a more than 1.5 folds of change in differentially expressed circRNAs (Fig. S1A). The scatter plots demonstrated 98 up-regulated and 40 down-regulated circRNAs in the plasma samples of PCa patients compared to the control individuals (Fig. S1B). Among them, the top 20 dysregulated circRNAs were listed in Fig. 1A. We focused on the top 5 exonic circRNAs with up-regulated expression, namely: circMAPKBP1, circRAN, circAHI1, circRBM4 and circSMARCC1. Then, further detected the relative expression levels of 5 circRNAs by RT-PCR in 39 pairs plasma samples of PCa and BPH. The results showed that compared with the plasma derived from patients with BPH, the expression levels of circRAN and circAHI1 were not significantly different, while the expression levels of circMAPKBP1, circRBM4 and circSMARCC1 were up-regulated, with circSMARCC1 being the most significantly up-regulated (Fig. S1C). Therefore, we chose circSMARCC1 as the research target.

CircSMARCC1 (hsa_circ_0001296), which was formed from exons 14, 15, and 16 of the coding gene SMARCC1 by back-splicing on the basis of the annotation of circBase (http://www.circbase.org/).The melting curve of circSMARCC1 amplified product using divergent primers showed a single peak the same as GAPDH (Fig. S1D). Its back-splicing junction was validated by Sanger sequencing and the presence of circSMARCC1 was demonstrated by RT-PCR (Fig. 1B). We designed the divergent and convergent primers to amplify the circSMARCC1 circular transcripts and SMARCC1 linear transcripts. PCR results showed that circSMARCC1 was only detected in cDNA, thus ruling out the existence of circSMARCC1 in gDNA, whereas the convergent primers amplified SMARCC1 from both cDNA and gDNA (Fig. 1C). To clarify the subcellular localization of circSMARCC1, we performed nuclear-cytoplasmic fractionation and FISH experiments in PCa cells and tissues. It was found that circSMARCC1 was mainly present in the cytoplasm of PCa cells (Fig. 1D). Additionally, nuclear-cytoplasmic fractionation assays showed that circSMARCC1 was mainly localized in the cytoplasm of PCa cells by qRT-PCR (Fig. 1E). The Actinomycin D assay demonstrated that circSMARCC1 was more stable compared with linear SMARCC1 (Fig. 1F). Moreover, we found that circSMARCC1 was more resistant to RNase R digestion than linear SMARCC1 (Fig. 1G). These results suggested that circSMARCC1 exists as a circular form and is highly stable. Then, we predicted the function of the circSMARCC1 encoded protein by circRNADb (http://reprod.njmu.edu.cn/cgi-bin/circrnadb/circRNADb.php). The results indicated that no open reading frame was found, which means that the possibility of circSMARCC1 in encoding protein is low (Fig. S1E), implying that circSMARCC1 is not encoded protein.

### CircSMARCC1 is up-regulated in PCa and associated with clinical characteristics

**Fig. 2:**
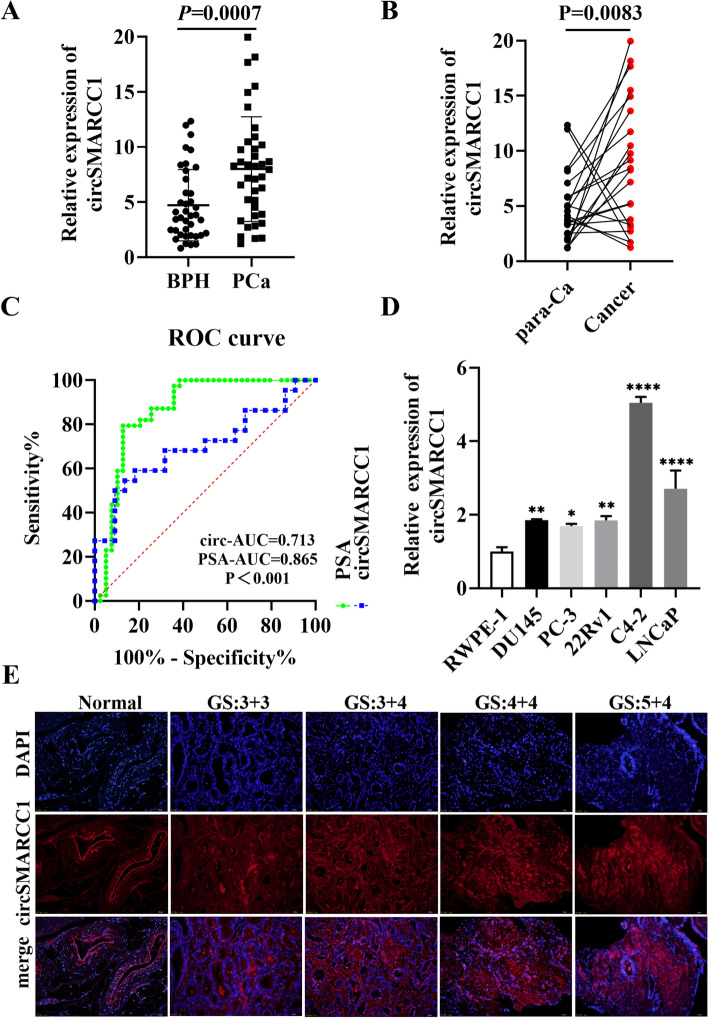
circSMARCC1 up-regulated in PCa and correlated with pathological parameters. **A**, **B** The relative expression of circSMARCC1 in plasma (*n* = 39) and tissue (*n* = 22) specimens from PCa patients and BPH patients was detected by qRT-PCR. **C** ROC curve analysis of the diagnostic value of circSMARCC1 and PSA for PCa. **D** The relative expression of circSMARCC1 in PCa cell lines by qRT-PCR. **E** The correlation between the expression of circSMARCC1 and Gleason score (GS) in PCa tissues through FISH experiment (scale bar, 20 μm). The data are presented as the mean ± SD, *p < 0.05, **p < 0.01, ***p < 0.001, ****p < 0.0001

To evaluate the expression and clinical value of circSMARCC1, qRT-PCR was carried out to detect the expression level of circSMARCC1 in the plasma of 39 pairs of age-matched patients with PCa and BPH. The results indicated that the expression of circSMARCC1 was significantly elevated in the plasma of PCa patients, which was consistent with the results of RNA-seq (Fig. 2A). Likewise, we validated the expression levels of circSMARCC1 in 22 pairs para-cancerous and PCa tissues. The results showed that circSMARCC1 was also significantly up-regulated in PCa tissues (Fig. 2B). Moreover, the diagnostic value of circSMARCC1 for PCa screening was further assessed utilizing the receiver operating characteristic (ROC) curve. Based on ROC curve analysis, the area under the curve (AUC) for circSMARCC1 was 0.713, with a specificity and sensitivity of 74.4 and 66.7%, respectively, and a cut-off value of 5.937 (Fig. 2C). It suggested that circSMARCC1 has the ability to identify PCa, but not as well as prostate specific antigen (PSA). Subsequently, we assessed the relationship between circSMARCC1 expression and clinicopathological features. The results showed that circSMARCC1 levels were positively correlated with Gleason score and T-stage in PCa patients, but not with age or PSA (Table 1).

**Table 1:** Correlation between circSMARCC1 expression and clinicopathological features in plasma from patients with BPH (*n* = 39) and PCa (*n* = 39)

Characteristic		N	circSMARCC1		χ2	*P*-value
			low	high		
Type	BPH	39	29	10	13.206	<0.001***
	PCa	39	13	26		
Age	≤ 67	21	8	13	0.464	0.496
	>67	18	5	13		
PSA	≤ 10	14	7	7	2.730	0.098
	>10	25	6	19		
Gleason score	ISUP ≤3	19	10	9	6.209	0.013*
	ISUP>3	20	3	17		
T stage	T1–2	22	11	11	6.309	0.012*
	T3–4	17	2	15		

*Abbreviations*: *PCa* Prostate cancer, *BPH* Benign prostatic hyperplasia, *PSA* Prostate specific antigen, *p < 0.05, ***p < 0.001

Furthermore, the expression of circSMARCC1 was validated in PCa tissues and cell lines. We analyzed the endogenous expression of circSMARCC1 by qRT-PCR. The results demonstrated that the expression of circSMARCC1 is higher in five PCa cell lines (PC-3, DU145, 22Rv1, C4–2 and LNCaP) than in the normal prostate epithelial cell line (Fig. 2D). Meanwhile, we examined the expression of circSMARCC1 in PCa tissues by RNA-FISH. The results showed that circSMARCC1 was significantly up-regulated in PCa tissues, and down-regulated in normal adjacent tissues (Fig. 2E). More importantly, it was clear that as the Gleason score of PCa increased, the expression of circSMARCC1 was also significantly up-regulated. In addition, using publicly available sequencing data for circRNAs [21], we validated the prognostic relationship between circSMARCC1 and biochemical recurrence (BCR) in PCa patients. We identified a trend that PCa patients with high circSMARCC1 expression were more likely to experience BCR early after radical prostatectomy, implying that high circSMARCC1 expression indicates a poorer prognosis, although we did not observe a statistically significant difference (Fig. S1F). These results suggested that circSMARCC1 might act as a tumor promoter, and high expression of circSMARCC1 could be a predictor of poor prognosis in PCa patients.

### CircSMARCC1 accelerates PCa cell proliferation, migration, and invasion in vitro

**Fig. 3:**
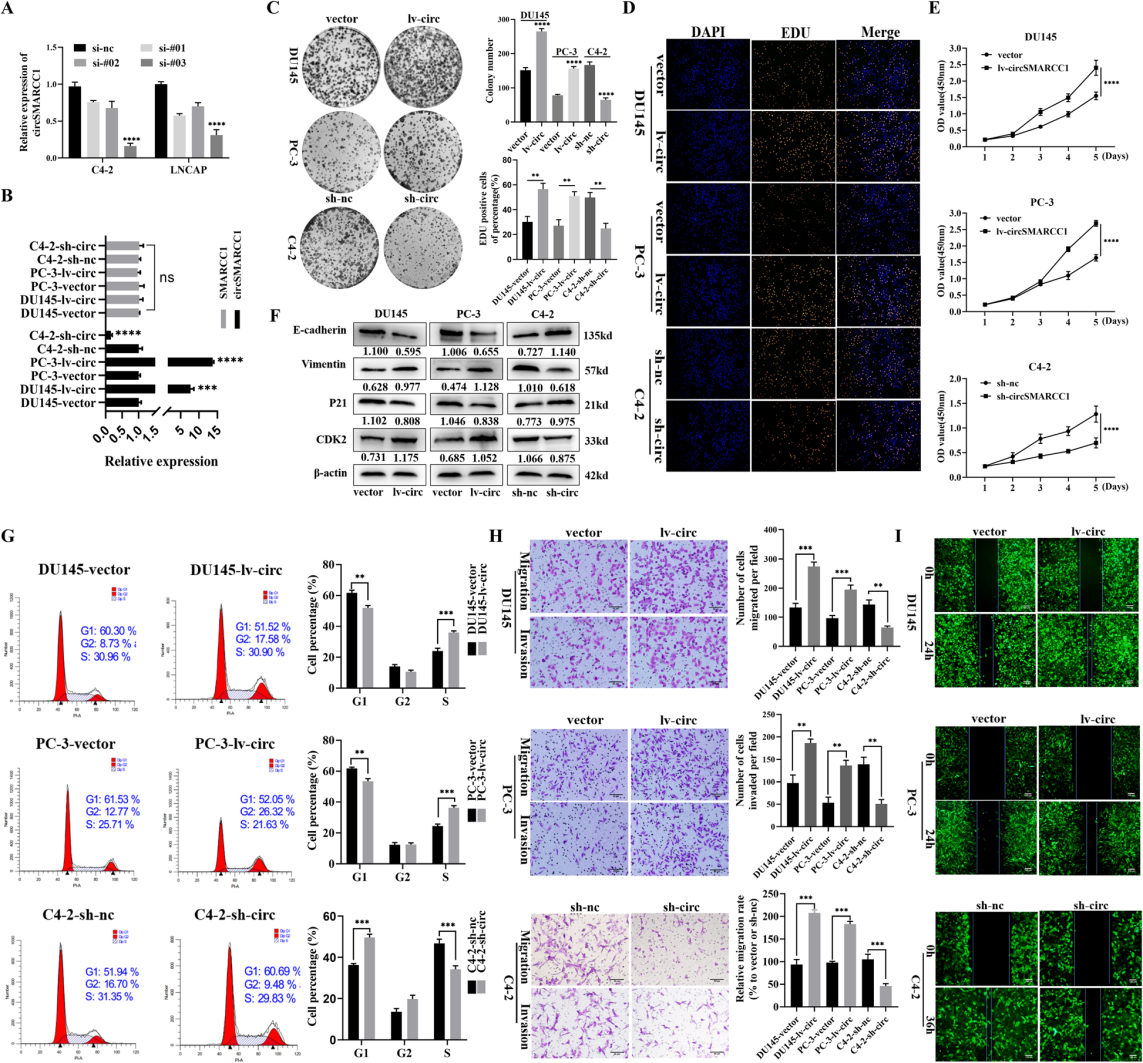
CircSMARCC1 promotes proliferation, migration and invasion of PCa cells. **A** three siRNAs (si-#01,02,03) targeting circSMARCC1 were constructed to silence its expression and confirmed by qRT-PCR. **B** The relative expression of circSMARCC1 and SMARCC1 in PCa cells transfected with circSMARCC1 overexpressing or knockdown lentivirus by qRT-PCR. **C-E** Assessment of cell proliferation capacity by colony formation, EdU assay (scale bar, 100 μm) and CCK-8 assay. **F** Western blot analysis evaluated expression of cell cycle-associated proteins and EMT biomarkers following overexpression or knockdown of circSMARCC1. **G** Flow cytometric analysis of changes in the cell cycle profile of PCa cells stably transfected with circSMARCC1. **H**, **I** Transwell assays and wound healing assays assessed the migration and invasion abilities of PCa cells stably transfected with circSMARCC1 (Scalebar, 50 μm). The data are presented as the mean ± SD, **p < 0.01, ***p < 0.001, ****p < 0.0001

As C4–2 cells exhibited the highest levels of circSMARCC1, and DU145 and PC-3 cells expressed lower levels of circSMARCC1 compared to other cells, we selected DU145, PC-3, and C4–2 cells for the subsequent studies. To clarify the potential role of circSMARCC1 in promoting PCa progression, three siRNAs (si-#01,02,03) targeting circSMARCC1 were constructed to silence its expression. The si-#03 exhibited the highest silencing efficiency measured by qRT-PCR and was chosen for the following experiments (Fig. 3A). We up-regulated circSMARCC1 expression in DU145 and PC-3 cells and down-regulated circSMARCC1 in C4–2 cells using a lentiviral vector and confirmed no change of SMARCC1 expression in parental cells (Fig. 3B). The colony formation, EdU, and CCK-8 assays were used to detect cell proliferation and viability. The results revealed that knockdown of circSMARCC1 significantly inhibited the proliferative capacity of PCa cells, while overexpression of circSMARCC1 resulted in increased cell viability (Fig. 3C-E). Furthermore, flow cytometry analysis was performed to confirm whether changes in proliferation were attributed to alterations in the cell cycle profile. The results showed that overexpression of circSMARCC1 in PC-3 and DU145 cells accelerated cell cycle progression at the S phase, whereas knockdown of circSMARCC1 in C4–2 cells induced G1 phase arrest (Fig. 3G). In addition, Western blot analysis showed that down-regulation of circSMARCC1 decreased CDK2 expression and increased p21 Waf1/Clip1 levels, while up-regulation of circSMARCC1 showed the opposite results (Fig. 3F). These results suggested that circSMARCC1 enhances cell growth, at least partially by inducing the G1/S transition in PCa cells. Similarly, we found that down-regulation of circSMARCC1 suppressed the migratory and invasive capacity of PCa cells through transwell and wound healing assays, whereas up-regulation of circSMARCC1 exhibited the opposite effect (Fig. 3H-I). Meanwhile, Western blot analysis showed that overexpression of circSMARCC1 distinctly reduced E-cadherin expression and increased Vimentin levels, while silencing of circSMARCC1 revealed the opposite effect (Fig. 3F). These findings indicated that circSMARCC1 has an oncogenic role in PCa cells.

### CircSMARCC1 directly binds to miR-1322 and suppresses miR-1322 activity

**Fig. 4:**
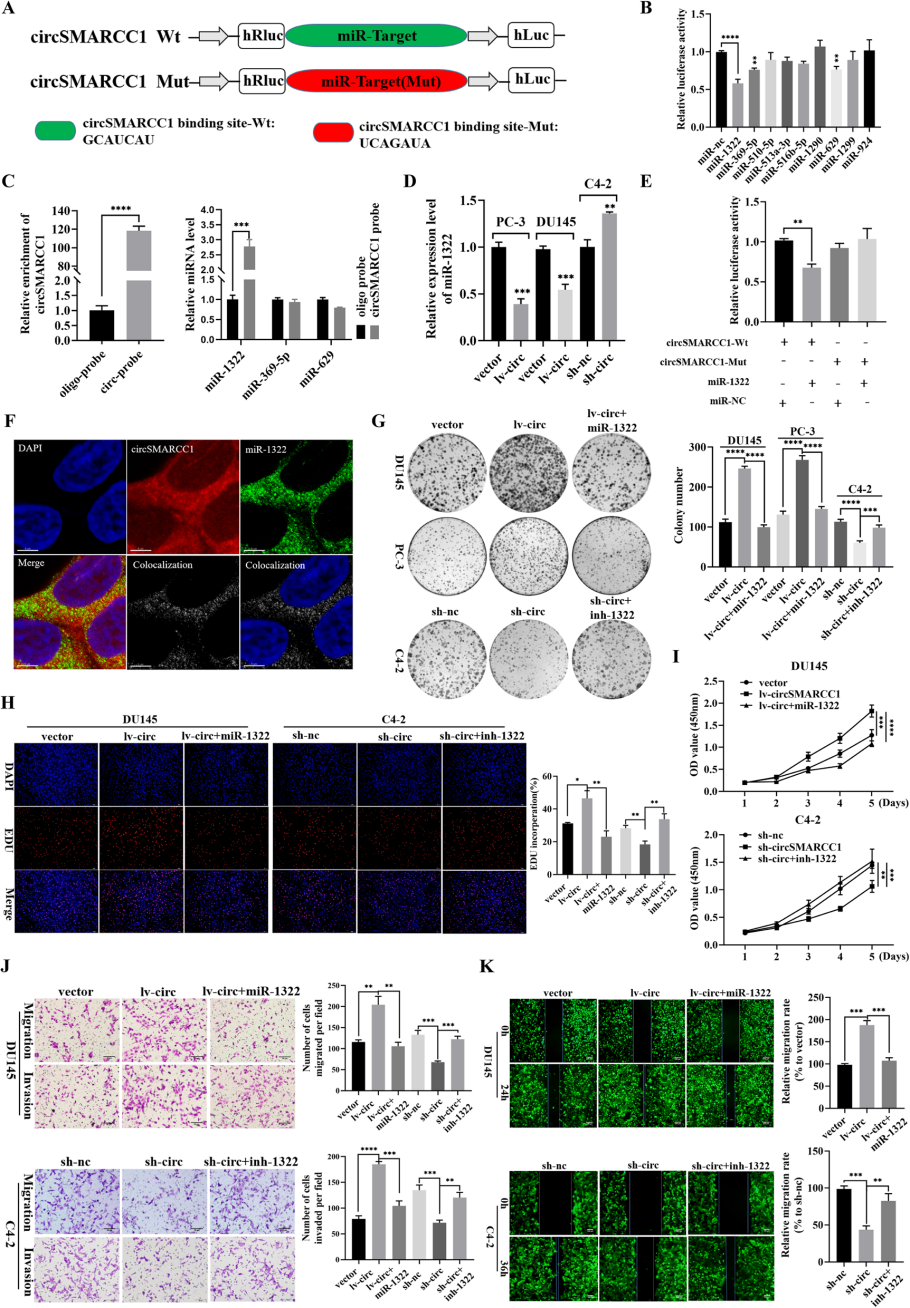
CircSMARCC1 acts as a sponge for miR-1322 and miR-1322 reverses the oncogenic effects of circSMARCC1 on proliferation, invasion and migration in PCa cells. **A** Schematic diagram of circSMARCC1 luciferase reporter vectors carrying wild-type (Wt) or mutant (Mut) miR-1322 binding sites. **B** Luciferase reporter assay to analyze the effects of 9 candidate miRNAs on the luciferase activity of circSMARCC1. **C** The RNA pull-down assay performed in DU145 cells using circSMARCC1 and negative control probes. **D** The relative expression of miR-1322 in PCa cells after transfection of circSMARCC1 was detected by qRT-PCR. **E** The relative luciferase activities measured in 293 T cells co-transfected with circSMARCC1-Wt or circSMARCC1-Mut and miR-1322 mimics or miR-nc by luciferase reporter assay. **F** The co-localization of circSMARCC1 and miR-1322 observed using RNA-FISH in DU145 cells (scale bar, 5 μm). The nuclei were stained with DAPI. **G-I** The viability of PCa cells in miR-1322 rescue experiments was analyzed by colony formation assay, EdU assay (scale bar, 100 μm) and CCK8 assay, respectively. **J**, **K** The migration and invasion capacity of PCa cells in the miR-1322 rescue experiment was analyzed by transwell assays (scale bar, 50 μm) and wound healing (scale bar, 100 μm) assays. The data are presented as the mean ± SD, *p < 0.05, **p < 0.01, ***p < 0.001, ****p < 0.0001

Endogenous circRNAs have been found to act as microRNA (miRNA) sponges in human cancers. As circSMARCC1 is mainly localized in the cytoplasm, we hypothesized that circSMARCC1 could regulate the expression of downstream molecules by binding to specific miRNAs. To investigate whether circSMARCC1 could sponge miRNA in PCa cells, we selected the 9 top potential miRNAs with a score ≥ 90 predicted by the CircInteractome database (Supplementary Table S4). To confirm the interaction between circSMARCC1 and the candidate miRNAs, dual-luciferase reporter assays were performed to detect the binding between circSMARCC1 and miRNAs. The wild-type and mutant dual-luciferase reporter plasmids of circSMARCC1 were constructed (Fig. 4A). The luciferase-circSMARCC1 reporters were transfected into HEK-293 T cells along with miRNA mimics or negative controls, and the data indicated that miR-1322 significantly reduced luciferase activity (Fig. 4B). Subsequently, RNA pull-down experiments were performed using biotinylated circSMARCC1 probes. The results demonstrated that miR-1322 was substantially pulled down by the biotin-coupled circSMARCC1 probe rather than the oligo probe in DU145 cells with circSMARCC1 overexpression, suggesting that circSMARCC1 might directly binds to miR-1322 (Fig. 4C). Besides, qRT-PCR assays were performed to investigate the effect of circSMARCC1 on miR-1322 expression. The results revealed that overexpression or knockdown of circSMARCC1 resulted in down-regulation or up-regulation of miR-1322 in PCa cells (Fig. 4D), while the expression of circSMARCC1 after transfection with miR-1322 mimics or inhibitors shows no significant changes (Fig. S2A). Furthermore, luciferase reporter assays were conducted in DU145 cells that were transfected with the wild-type and mutant dual-luciferase reporter plasmids of circSMARCC1. The luciferase activity was obviously decreased in cells co-transfected with the miR-1322 mimics and the circSMARCC1-Wt luciferase reporter when compared with the mutants (Fig. 4E). In addition, RNA-FISH was utilized to confirm the subcellular co-localization of circSMARCC1 and miR-1322 in PCa cells. We found that circSMARCC1 co-localized with miR-1322 in the cytoplasm (Fig. 4F). These results demonstrated that circSMARCC1 could directly bind to miR-1322 in PCa cells.

### MiR-1322 reverses the tumor-promoting effect of circSMARCC1 in PCa cells

To determine whether circSMARCC1-mediated miR-1322 affected the cell viability as well as migration and invasion of PCa cells, several rescue experiments by co-transfection of miR-1322 mimics or miR-1322 inhibitors with lv-circSMARCC1 or sh-circSMARCC1 were performed. The results showed that ectopic expression of miR-1322 significantly attenuated the proliferation, migration and invasion promotion induced by circSMARCC1 up-regulation, while miR-1322 inhibitor counteracted the inhibitory effect of circSMARCC1 down-regulation in proliferation, migration and invasion by colony formation assays (Fig. 4G), EdU analysis (Fig. 4H), CCK8 experiments (Fig. 4I), transwell assays (Fig. 4J) and wound healing assays (Fig. 4K). Overall, these experiments suggested that circSMARCC1 serves as a sponge for miR-1322.

### CCL20 is a direct target of miR-1322 and activates PI3K-Akt pathway through circSMARCC1/miR-1322/CCL20 axis

**Fig. 5:**
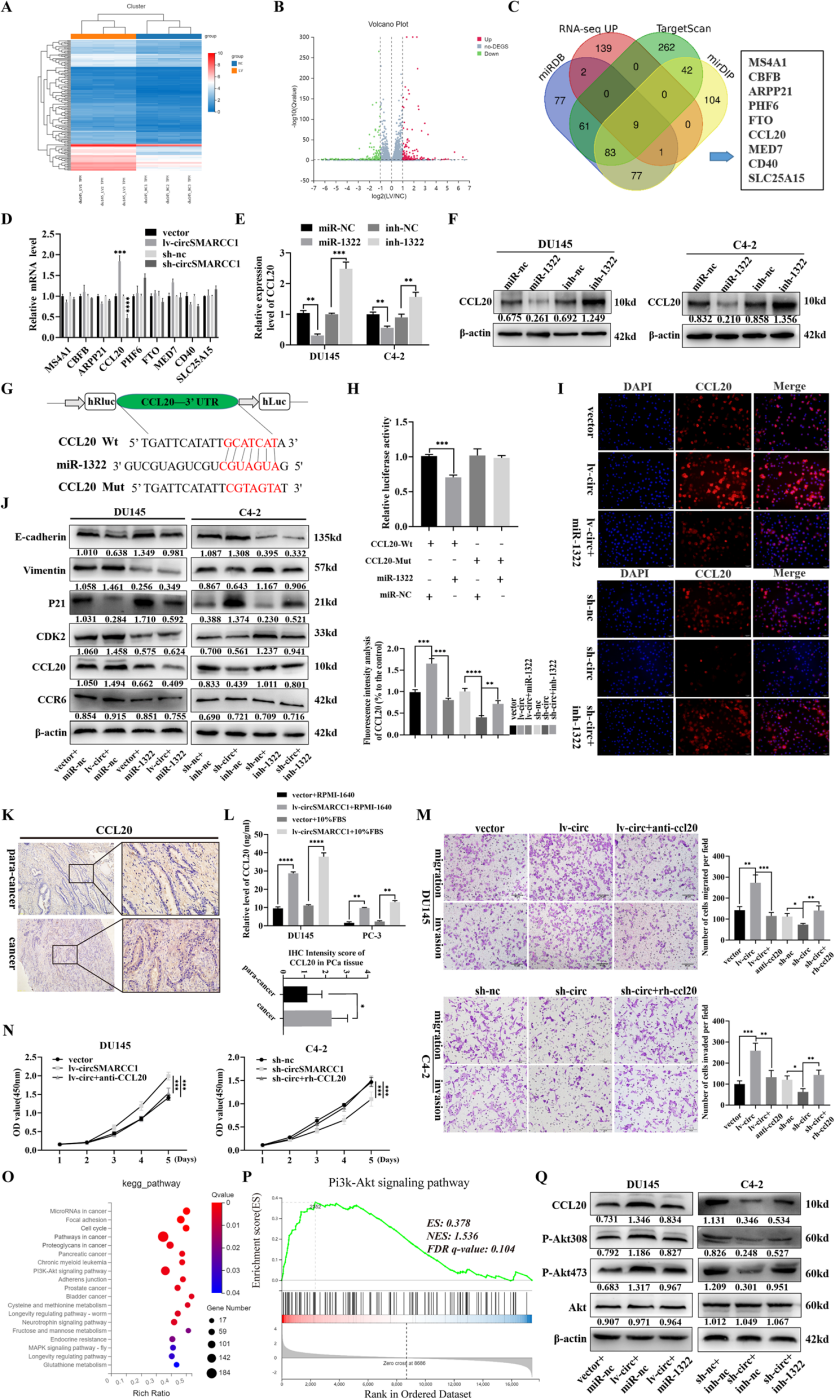
CCL20 is a direct target of miR-1322 and activates PI3K-Akt pathway through circSMARCC1/miR-1322/CCL20 axis. **A**, **B** The heatmap and volcano plot of differentially expressed mRNAs in DU145 cells transfected with vector or circSMARCCC1. Each sample was mixed with three replicates. **C** Venn diagram illustrating the overlapping of miR-1322 targeting mRNAs and up-regulated mRNAs in DU145 cells. **D** qRT-PCR was performed to detect the mRNA expression of 9 candidate molecules (MS4A1, CBFB, ARPP21, PHF6, FTO, CCL20, MED7, CD40 and SLC25A15). **E**, **F** The expression of CCL20 in PCa cells transfected with miR-1322 mimics or miR-1322 inhibitors was detected by qRT-PCR and Western blotting. **G** Schematic illustration of CCL20 wide type (Wt) and mutant (Mut) luciferase reporter vectors were shown. **H** The relative luciferase activities measured in 293 T cells co-transfected with CCL20-Wt or CCL20-Mut and miR-1322 mimics or miR-nc by luciferase reporter assay. **I** IF analysis detected the protein expression of CCL20 in PCa cells transfected or co-transfected with lv-circSMARCC1, sh-circSMARCC1, miR-1322 mimics or inhibitors (scale bar, 50 μm). **J** The relative protein levels of CCL20, EMT biomarkers and cell cycle-related molecules detected by Western blotting in miR-1322 rescue experiments. **K** IHC detection of CCL20 expression in PCa tissues and para-cancerous tissues (scale bar, 100 μm and 20 μm). **L** the Secretion of CCL20 in the supernatants of circSMARCC1-overexpressing DU145 and PC-3 cells cultured in RPMI-1640 medium or 10% FBS medium was measured by ELISA. **M**, **N** The CCL20 recombinant and CCL20 neutralizing antibodies were applied to transwell assays and CCK8 assays to explore the effects of CCL20 on PCa cell proliferation, migration and invasion. **O**, **P** KEGG and GSEA analyses of DEGs in DU145 cells. **Q** The relative protein levels of CCL20 and AKT pathway-related molecules measured by Western blotting in miR-1322 rescue experiments. The data are presented as the mean ± SD, *p < 0.05, **p < 0.01, ***p < 0.001, ****p < 0.0001

To further explore the underlying mechanism, we performed RNA-seq in DU145-lv-circSMARCC1 and DU145-vector cells and found that 151 genes were up-regulated and 209 genes were down-regulated in DU145-lv-circSMARCC1 cells compared to DU145-vector cells (fold change > 2 and *p* < 0.05). The cluster heat map and volcano plots of the differential genes were shown in Fig. 5A, B. Subsequently, we conducted bioinformatics analyses using Targetscan (http://www.targetscan.org), miRDB (http://www.mirdb.org/) and miRDIP (https://ophid.utoronto.ca/mirDIP/index.jsp) to predict possible downstream targets for miR-1322 binding. Combined with the results of RNA sequencing, the data showed that 9 molecules containing conserved target sites of miR-1322 may serve as downstream targets of mir-1322 (Fig. 5C). Next, we used qRT-PCR to verify the 9 candidate molecules. The results showed that CCL20, but not other candidate targets, was up-regulated in DU145-lv-circSMARCC1 cells and down-regulated in C4–2-sh-circSMARCC1 cells (Fig. 5D). Further, we successfully constructed miR-1322-overexpressing and miR-1322-knockdown cells transfected with miR-1322 mimics or miR-1322 inhibitors (Fig. S2B). As shown in Fig. 5E-F, CCL20 expression was significantly down-regulated or up-regulated at both mRNA and protein levels in PCa cells transfected with miR-1322 mimics or miR-1322 inhibitors. Furthermore, wild-type and mutant dual luciferase reporter plasmids of CCL20 were constructed (Fig. 5G). Using a dual luciferase reporter assay, we found that transfection of miR-1322 mimics can significantly reduce the activity of the wild-type luciferase reporter gene, but not the mutant of CCL20 (Fig. 5H). These data suggested that miR-1322 directly targets CCL20.

To explore whether circSMARCC1/miR-1322 affects CCL20 expression, immunofluorescence (IF) and Western blot analysis were performed on lv-circSMARCC1 and sh-circSMARCC1 tumor cells. The results showed that lv-circSMARCC1 remarkably increased CCL20 expression, while sh-circSMARCC1 distinctly decreased CCL20 levels. Furthermore, miR-1322 mimics or inhibitors could reverse the increase or decrease of CCL20 induced by circSMARCC1 overexpression or knockdown (Fig. 5I-J). Importantly, we further found that CCL20 was up-regulated in PCa tissues compared with para-cancer tissues via IHC assay (Fig. 5K). Since CCL20 is a secreted protein, we also detected a significant increase in CCL20 secretion in the supernatants of DU145 and PC-3 cells overexpressing circSMARCC1 by ELISA, both in RPMI-1640 medium and in 10% FBS medium (Fig. 5L). These data suggested that CCL20 is a direct downstream of miR-1322.

As reported, CCL20 induces EMT in ovarian cancer cells and contributes to tumor progression [22]. We further assessed whether CCL20 could enhance the proliferative and metastatic capacity of PCa cells. CCL20 was found to be significantly up-regulated in PCa cells (Fig. S2C). We designed three small interfering RNAs (siRNAs) targeting CCL20 (si-#01, 02, 03), and found that si-#01 had the highest knockdown efficiency and used it for follow-up studies (Fig. S2D). The CCK8 experiments indicated that knockdown of CCL20 significantly down-regulated the proliferation ability of cells (Fig. S2E). Meanwhile, transwell assay showed that downregulation of CCL20 significantly reduced the number of migrating PCa cells (Fig. S2F). We then applied CCL20 recombinant protein and CCL20 neutralizing antibody to transwell assays and CCK8 assay. The results showed that CCL20 neutralizing antibody impaired the metastasis and proliferation caused by overexpression of circSMARCC1, and CCL20 recombinant protein reversed the reduction in metastasis and proliferation caused by knockdown of circSMARCC1(Fig. 5M, N). Furthermore, we interpreted those events in terms of changes in cell cycle proteins and EMT-related proteins by Western blotting. It was found that up-regulation of circSMARCC1 increased Vimentin and CDK2 expression and decreased E-cadherin and p21 Waf1/Clip1 expression, while down-regulation of circSMARCC1 had the opposite effects. Importantly, these effects could be abolished by miR-1322 mimics or inhibitors, respectively (Fig. 5J).

In addition, we examined the downstream signal pathways to which the miR-1322/CCL20 axis might transmit signals. We performed pathway analysis (KEGG and GSEA) based on RNA-seq profiles and found that overexpression of circSMARCC1 was positively correlated with the PI3K-Akt signaling pathway (Fig. 5O, P). To confirm this result, further studies showed that overexpression of circSMARCC1 increased the expression of CCL20, P-Akt473 and P-Akt308, while knockdown of circSMARCC1 exerted the opposite effect. Similarly, these effects could be eliminated by miR-1322 mimics or inhibitors (Fig. 5Q). Furthermore, western blot analysis showed that knocking down CCL20 down-regulated the expression of P-Akt473 and P-Akt308 (Fig. S2G). Finally, to further confirm whether the PI3K-Akt was required for circSMARCC1-mediated promotion of PCa progression, we performed rescue experiments by using LY294002, a PI3K-Akt inhibitor. The results showed that LY294002 could eliminate the increased cell proliferation (Fig. S2H, I) and migration (Fig. S2J) due to overexpression of circSMARCC1. In addition, Western blotting analysis was conducted to evaluate the effect of treatment with LY294002 on the levels of related proteins. Our results demonstrated that LY294002 partially restored p21 Waf1/Clip1 and E-cadherin expression while decreased Vimentin and CDK2 levels in PCa cells with ectopic expression of circSMARCC1 (Fig. S2K). These results suggested that circSMARCC1 functions as sponge of miR-1322 to promote PCa progression via activating Akt pathway.

### CircSMARCC1 promotes PCa cells growth and metastasis in vivo

**Fig. 6:**
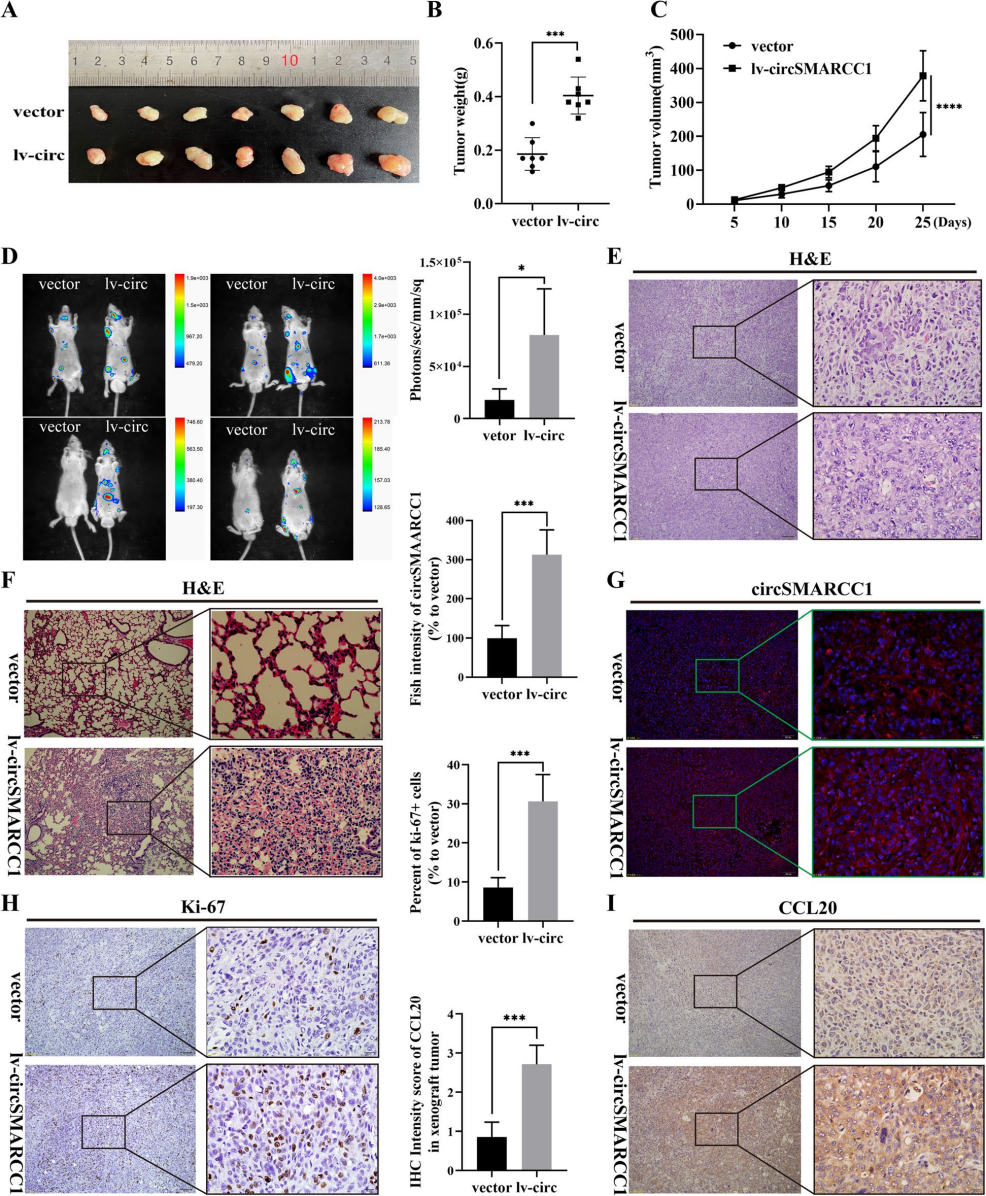
circSMARCC1 promotes PCa tumorigenesis and metastasis in vivo. **A** Images of subcutaneous xenograft tumors derived from DU145 cells transfected with vector and circSMARCC1. **B** Tumor weight comparison in lv-circ group and vector group. **C** Tumor volumes measured once a week and the growth curves were drawn. **D** Fluorescence image of metastatic transplanted tumor from PC-3 cells transfected with vector or circSMARCC1. **E** H&E staining of subcutaneous xenograft tumors in mice (scale bar, 100 μm and 20 μm). **F** H&E staining of metastatic lung tumors in mice (scale bar, 100 μm and 20 μm). **G** RNA-FISH experiment showed the fluorescence area in xenograft tumor tissues of vector and lv-circSMARCC1 groups (scale bar, 100 μm and 20 μm). **H**, **I** The expression of Ki-67 and CCL20 in xenograft tumors detected by IHC (scale bar, 100 μm and 20 μm). *p < 0.05, ***p < 0.001, ****p < 0.0001

To investigate the biological function of circSMARCC1 in vivo, we constructed a xenograft tumor model by injecting circSMARCC1-overexpressing or vector DU145 cells into the axilla of BALB/c mice (Fig. 6A). The results showed heavier tumors and faster tumor growth in the overexpression group compared to the vector group (Fig. 6B, C). To study the effect of metastasis, a metastatic tumor model was generated by tail vein injection of nude mice using circSMARCC1-overexpressing or vector PC-3 cells. The PC-3 cells were pre-labelled with the luciferase gene to facilitate observation of tumor metastases in vivo by bioluminescence. We observed that mice in the overexpression group developed more lung and abdominal metastases compared to the vector group (Fig. 6D and S2L). H&E staining shows the pathological features of subcutaneous tumor tissue and isolated pulmonary metastases foci (Fig. 6E, F). Besides, RNA-FISH experiments in tumors showed a more extensive fluorescent area in the xenograft tumor tissue of lv-circSMARCC1, confirming that circSMARCC1 expression was indeed up-regulated (Fig. 6G). The up-regulation of circSMARCC1 expression was accompanied by an increase in the proportion of proliferating cells (Ki67+) via IHC experiments (Fig. 6H). These results suggested that circSMARCC1 promotes the growth of PCa in vivo. Next, IHC results showed a significant up-regulation of CCL20 expression in the lv-circSMARCC1 xenograft tumor group, which is consistent with the results we observed in human PCa tissues (Fig. 6I). In addition, we examined the expression of CD31 in tumor tissue, and found that CD31 expression was up-regulated in the lv-circSMARCC1 group, indicating active tumor angiogenesis (Fig. S2M). These findings demonstrated that circSMARCC1 promoted PCa growth and metastasis in vivo.

### Overexpression of circSMARCC1 increases the number of M2 macrophages in human PCa tissues and mouse allograft tumors

**Fig. 7:**
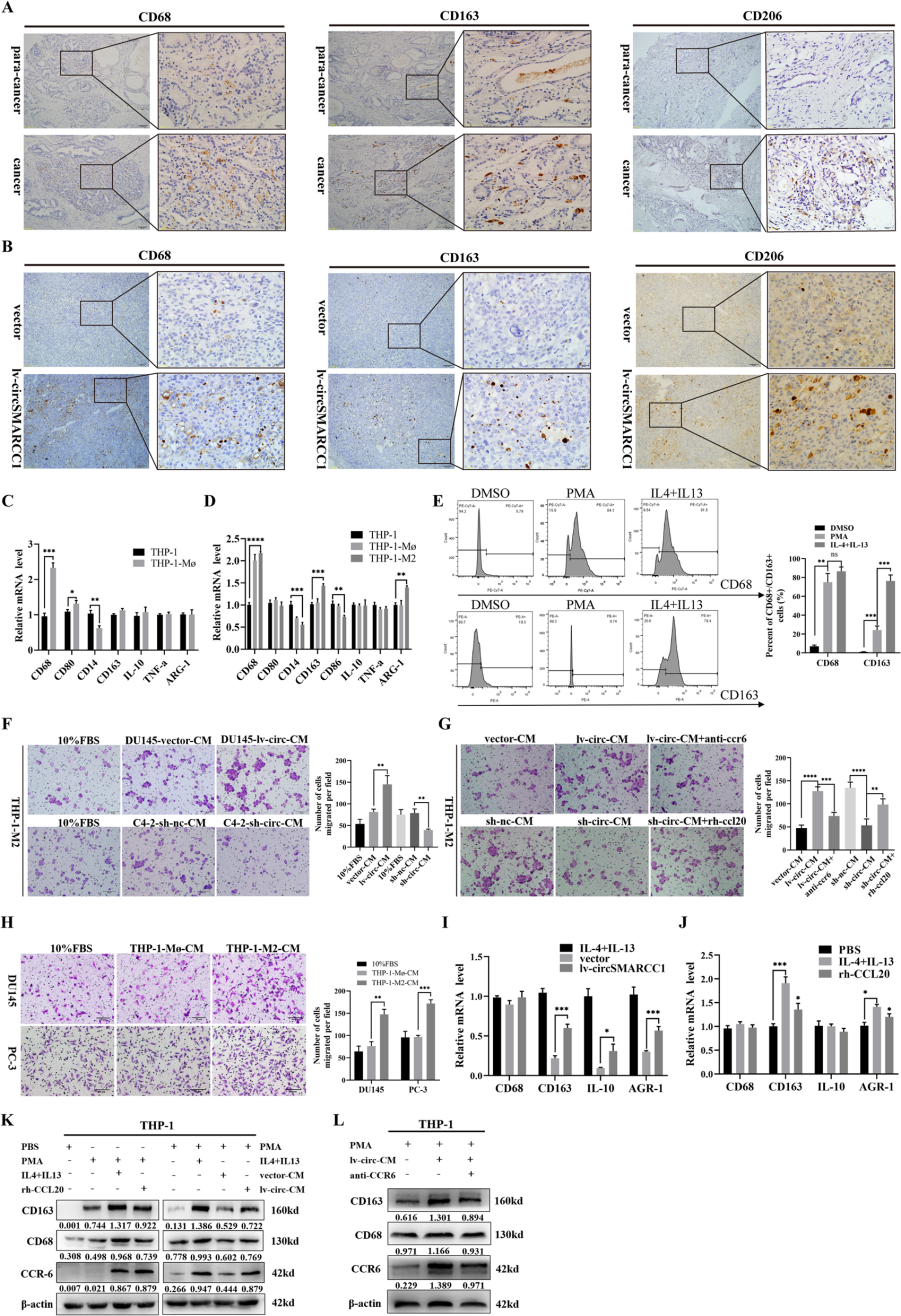
circSMARCC1 promotes M2 macrophage polarization and recruitment via the CCL20-CCR6 axis. **A** IHC detected the infiltration of CD68^+^/ CD163^+^/ CD206^+^ macrophages in human PCa tissue and Para-cancerous tissues (scale bar, 100 μm and 20 μm). **B** IHC detected the infiltration of CD68^+^/ CD163^+^/ CD206^+^ macrophages in mouse xenograft tumors (scale bar, 100 μm and 20 μm). **C**, **D** The expression of M1 phenotype (TNF-a, CD80 and CD86) and M2 phenotype (IL-10, ARG-1 and CD163) markers were detected by qRT-PCR in THP-1 cells, THP-1-Mø and THP-1-M2 cells. **E** The proportion of CD68 and CD163 positive cells detected by flow cytometry. **F** The CM prepared from lv-circSMARCC1 or vector and sh-circSMARCC1 or sh-nc tumor cells was used to evaluate the migration ability of macrophages through transwell experiments (scale bar, 50 μm). **G** The migration ability of TAM was detected by transwell assay using CCL20 recombinant protein and CCR6 neutralizing antibody (scale bar, 50 μm). **H** The CM prepared from THP-1-Mø or THP-1-M2 cells for PCa cells migration experiments (scale bar, 50 μm). **I**, **J** qRT-PCR tested the expression of M2 phenotypes marker (IL-10, ARG-1, CD68 and CD163) when co-cultured with DU145-lv-circSMARCC1 cells or vector cells or using CCL20 recombinant protein, with IL-4 and IL-3 polarization as the reference. **K** The expression of CD68, CD163 and CCR6 in THP-1 cells, THP-1-Mø cells stimulated by CCL20 recombinant protein, and THP-1-Mø cells (with and without co-culture) were detected by Western blotting. **L** The expression of CD68, CD163 and CCR6 in THP-1-Mø cells and THP-1-M2 cells (with and without anti-CCR6) were detected by Western blotting. *p < 0.05, **p < 0.01, ***p < 0.001, ****p < 0.0001

Chemokines regulate tumor progression in the TME through chemotaxis of macrophages or immune cells [23]. To further assess the role of CCL20, we analyzed the differential function of CCL20 as well as CCL20 co-expressed genes in PCa from TCGA data using Gene Ontology (GO) analysis. The results showed that the most differentially expressed genes were associated with immune system processes (Fig. S3A). Next, the correlation between CCL20 and 28 tumor-infiltrating lymphocytes types in the TME of PCa was analyzed using the TISIDB database (http://cis.hku.hk/TISIDB/). The results showed a significant positive correlation between CCL20 and macrophages (Fig. S3B). Similarly, we found that CCR6, the specific receptor for CCL20, also had a significant positive correlation with macrophages and was more closely related to M2-type macrophages among them (Fig. S3C). These evidences support our exploration of the relationship between the CCL20-CCR6 axis and macrophages in the TME of PCa. In addition, several studies have shown that CCL20 regulates macrophage recruitment to drive tumor growth in colon cancer [24] and to promote breast tumorigenesis [25]. Kfoury et al. described the PCa bone metastases and revealed an immune mechanism of M2 macrophage infiltration mediated by the CCL20-CCR6 axis [26]. Accordingly, we tested circSMARCC1 expression and macrophage infiltration by IHC in human PCa tissues and adjacent tissues. Interestingly, the number of TAMs (CD68^+^, CD163^+^ and CD206^+^) in PCa tissues with circSMARCC1 up-regulation was significantly higher than that in adjacent tissues with circSMARCC1 down-regulation (Fig. 7A). Furthermore, we obtained consistent results in BALB/c mice xenograft tumors. The proportion of CD68^+^/CD163^+^/CD206^+^ positive cells in xenograft tumors was significantly increased in the mice overexpressing circSMARCC1 group compared to the vector group (Fig. 7B). These results suggested that the expression of circSMARCC1 is positively correlated with M2 macrophage infiltration, and circSMARCC1 may induce M2 macrophage colonization.

### CircSMARCC1 promotes M2 macrophage recruitment through CCL20-CCR6 axis

The above results indicated that the expression levels of circSMARCC1 and CCL20 were positively correlated both in PCa cells and tissues. We hypothesized that circSMARCC1 could regulate M2 macrophage recruitment through the CCL20-dependent process. We treated THP-1 cells with 100 ng/ml PMA for 24 h and found that THP-1 cells became adherent and extended their pseudopod. qRT-PCR was performed to detect macrophage markers and we found that the expression of CD68, CD80 were significantly up-regulated and CD14 was down-regulated in THP-1 cells after PMA treatment compared to the control (Fig. 7C). The data suggested that THP-1 cells were induced to become THP-1-Mø. Next, THP-1-Mø were incubated with 20 ng/ml IL-4 and 20 ng/ml IL-13 for 48 h, and then the typical M1 phenotypes (TNF-a, CD80, and CD86) and M2 phenotypes (IL-10, ARG-1, and CD163) markers were investigated using qRT-PCR. The results showed a significant increase in mRNA of ARG-1 and CD163 expression stimulated by IL-4 and IL-13, while CD86 expression was down-regulated (Fig. 7D). In addition, flow cytometry demonstrated a substantial increase in the proportion of CD68^+^ and CD163^+^ macrophages (Fig. 7E). These results indicated a polarization of THP-1 towards THP-1-M2.

To investigate the effect of circSMARCC1 on the migration of TAMs, the CM prepared from lv-circSMARCC1 or vector and sh-circSMARCC1 or sh-nc tumor cells were added to the lower chamber for macrophage migration experiments. Consistent with the expected results, the number of migrating TAMs increased significantly when induced with the CM from lv-circSMARCC1 tumor cell, whereas TAM migration was significantly reduced in sh-circSMARCC1 group (Fig. 7F). CC-chemokine ligand 20 (CCL20) and its selective receptor CCR6 [27], known to be responsible for the chemoattraction of TAMs in homeostatic conditions [28], have been recently found to be involved in metastatic progression of colon cancer [24]. we hypothesized that the chemotaxis of TAMs may be due to the binding of CCL20 to CCR6 in PCa. By analyzing the TCGA database, we found a strong positive correlation between CCL20 and CCR6 levels in PCa tissues (Fig. S3D). To determine whether the fact that circSMARCC1 induces migration of TAMs is critically mediated by CCL20-CCR6 axis, we performed transwell migration assays using CCL20 recombinant protein and CCR6 neutralizing antibody. The results showed that the CCL20 recombinant protein significantly promoted the migration of TAMs, however, this promotion was reversed by the introduction of the CCR6 neutralizing antibody (Fig. 7G). These findings suggested that circSMARCC1 plays a key role in mediating the migration of M2 macrophages via CCL20-CCR6 axis.

TAM had been shown to promote EMT in tumor cells [29]. We prepared the CM from THP-1-Mø and THP-1-M2 separately and used them to perform migration assays in PCa cells. We observed that the CM of THP-1-M2 significantly increased the migration of DU145 and PC3 cells compared to the CM of THP-1-Mø (Fig. 7H). In addition, we also examined the effect of CM from THP-1-M2 on the proliferation of stably transfected PC cells. CCK8 results showed that the CM of TAMs did not alter the proliferation of PCa cells (Fig. S3E). These results suggested that circSMARCC1 enhances the recruitment of TAMs via the CCL20-CCR6 axis, thereby facilitating the progression of PCa.

### CircSMARCC1 promotes M2 macrophage polarization via the CCL20-CCR6 axis

To explore the effect of circSMARCC1 on the polarization of TAMs, we co-cultured the stably transfected PCa cells with THP-1-Mø. The PCa cells of lv-circSMARCC1 or vector, sh-circSMARCC1 or sh-nc were seeded in the 0.4 μm pore size upper inserts and then transferred to the 6-well plates pre-inoculated with THP-1-Mø. After 48 h, macrophages were collected for the following experiments. qRT-PCR test confirmed that the expression of M2 phenotype (IL-10, ARG-1 and CD163) markers was significantly up-regulated in the co-cultured with DU145-lv-circSMARCC1 cells compared with the vector cells. This was consistent with the results induced by IL-4 and IL-13 (Fig. 7I). We then stimulated THP-1-Mø using CCL20 recombinant protein alone and also observed an up-regulation of M2 phenotype markers (Fig. 7J). In addition, we examined changes in CD68, CD163 and CCR6 protein levels in THP-1 cells, THP-1-Mø and THP-1-Mø (with and without co-culture) by Western blotting. The results revealed that co-culture with DU145-lv-circSMARCC1 cells resulted in a significant up-regulation of CD163 expression in THP-1-Mø compared to the vector group. Moreover, addition of CCL20 recombinant protein alone was also able to induce an up-regulation of CD163 expression in THP-1-Mø, although not as pronounced as the up-regulation induced by IL-4 and IL-13(Fig. 7K). These data suggested that CCL20 plays a key role in M2 macrophage polarization and that CCL20 is able to partially induce M2 polarization in THP-1-Mø. Interestingly, we found that THP-1 cells and THP-1-Mø expressed no or little CCR6, but when they were polarized to THP-1-M2 by CCL20 recombinant or IL-4 and IL-13 stimulation, CCR6 expression was significantly up-regulated (Fig. 7K). Subsequently, we stimulated THP-1-Mø using IL-4 and IL-13 together with CCR6-neutralizing antibodies to achieve a blockade of CCL20 binding to the CCR6 receptor. We found that treatment with CCR6 neutralizing antibody significantly down-regulated the expression of CD163, but not CD68 (Fig. 7L). These data suggested that circSMARCC1 promotes M2 macrophage polarization in PCa via the CCL20-CCR6 axis.

## Discussion

The presence of circRNA in the cytoplasm of eukaryotic cells was first discovered by Hsu and Coca-Prados in 1979 [30]. For decades afterwards, circRNAs were thought to be the product of shearing errors. With the development of RNA-seq technology and bioinformatics, a large number of circRNAs have entered the scientific community. The extensive expression and disease regulation mechanisms of circRNAs have made them functional biomarkers and therapeutic targets for a variety of diseases. However, the role of circRNA in the progression of PCa is still not well studied.

Here, we investigated circRNA expression profiles in plasma samples from four pairs of mPCa patients and control patients using circRNA sequencing. We focused on a significantly differentially expressed novel circRNA, named circSMARCC1, which was significantly up-regulated in PCa and correlated significantly with clinical Gleason scores and T stage. Furthermore, a series of in vitro and in vivo experiments demonstrated that circSMARCC1 promoted PCa cell proliferation, migration and invasion, while knockdown of circSMARCC1 expression had the opposite effect.

Many circRNAs contain potential miRNA response elements, which suggests that circRNAs can act as miRNA sponges, forming a circRNA-miRNA-mRNA axis to exert their biological roles [31]. For instance, circASAP1 acts as a competing endogenous RNA (ceRNA), and binds to miRNAs as miRNA sponges in cells, which promotes hepatocellular carcinoma cell proliferation and invasion [32]. Hsa_circ_0000326 acts as a miR-338-3p sponge to facilitate lung adenocarcinoma progression [33]. In this study, we found that circSMARCC1 is highly expressed in PCa, especially in the cytoplasm of tumor cells. Therefore, we speculated that circSMARCC1 may also act as a miRNA sponge in PCa. Through bioinformatics analysis, luciferase assay and miRNA pulldown assays, we confirmed that circSMARCC1 serves as a sponge for miR-1322. Previous studies have reported that miR-1322 acts as tumor suppressors in some types of cancers, including hepatocellular carcinoma cells [34] and lung adenocarcinoma [35]. Consistent with that, our results confirmed that miR-1322 function as a tumor suppressor in PCa, which mediates the counteracts the oncogenic roles of circSMARCC1 on PCa proliferation and metastasis.

MiRNAs have been proved to play crucial roles in tumor cell differentiation, growth, and metastasis [36], which alleviating the inhibitory effects of their target genes [37]. In the present study, mRNA expression profiling, bioinformatics analysis and dual luciferase reporter gene assays revealed that CCL20 might act as direct targets of miR-1322. Further validation experiments demonstrated that circSMARCC1 may serve as an endogenous miRNA sponge to inhibit the expression of miR-1322 by binding to miR-1322 in PCa cells, resulting in alleviating the inhibitory effect of miR-1322 on CCL20 and ultimately promotes PCa cell proliferation and metastasis.

CCL20 (also known as macrophage inflammatory protein-3α, MIP-3α) is a pro-inflammatory chemokine. The CCL20 expression has been reported to be up-regulated in many cancers, such as hepatocellular carcinoma [38] and pancreatic cancer [39]. CCR6 is a specific receptor for CCL20, and CCR6 expression has also been shown on tumor cells. It was reported that tumor cells can stimulate their own proliferation and migration via CCL20-CCR6 in an autocrine manner [40–42]. Previous studies found that CCL20 can be a novel predictive marker for taxanes response, and blockade of CCL20 or its downstream pathway might reverse the taxanes resistance in breast cancer patients [43]. Whereas the specific role of CCL20 in PCa progression remains uncertain. Here, our study found that overexpression of circSMARCC1 increased the expression of CCL20, without changing the expression of its receptor CCR6 in PCa cells. Then, we confirmed that the up-regulation of CCL20 significantly accelerated the proliferation and metastasis of PCa cells, and that CCL20-neutralizing antibody reversed the above pro-tumorigenic effects. Therefore, our data indicates that circSMARCC1 sponges miR-1322 and up-regulates CCL20 to promote PCa progression in an autocrine manner.

Macrophages make up 30 to 50% of solid tumor-infiltrating immune cells, and they represent an important component of immunotherapy. In most cancers, high TAMs density is associated with poorer patient prognosis and treatment resistance. Macrophage depletion studies have shown great success in limiting tumor growth and metastatic spread and in restoring responsiveness to chemotherapy [44, 45]. An alternative to targeting TAM is to inhibit their recruitment to the primary tumor. CCL2 is a chemokine that regulates monocyte and macrophage migration, and the CCL2/CCR2 axis has been shown to have multiple pro-tumor effects, ranging from mediating tumor growth and angiogenesis to recruiting and usurping host stromal cells to support tumor progression [46]. In a phase 1 trial, the administration of a CCR2 inhibitor (PF-04136309) was well tolerated and showed promising anti-tumor activity in patients with advanced pancreatic cancer [47]. CXCL12 is also a chemokine that promotes migration of macrophages across the endothelial barrier into the tumor environment [48]. For this reason, inhibition of chemokines and their receptors signaling is a promising strategy for regulating macrophage infiltration and preventing tumor progression. In PCa, several studies reported that more TAM infiltration in the TME promoted PCa cell proliferation and migration and was associated with PSA failure or PCa progression after hormonal therapy [49, 50]. In fact, increased infiltration of TAMs often predicts poor prognosis in patients with mPCa [51, 52]. On the one hand, it has been reported that TAM-secreted CCL5 can promote PCa cell migration, invasion, and epithelial-mesenchymal transition (EMT) by activating β-catenin/STAT3 signaling [53]. On the other hand, high levels of cytokines and chemokines, including TGF-β, IL10 and CCL2 are secreted by PCa, which may contribute to the recruitment of various immunosuppressive cells (myeloid-derived suppressor cells and regulatory T cells) and tumor-promoting TAMs [54, 55], thereby promoting treatment tolerance and immune evasion by inhibiting CD4^+^ helper T (Th1) and CD8^+^ cytotoxic T cells [56]. Overall, these data suggest that TAMs play a dual role as “tumor promoter” and “immunosuppressant”, and targeting TAMs may represent a potential therapeutic strategy for mPCa.

Some noncoding RNAs, including circRNAs, have been shown to have strong effects on TAM in a variety of tumors, such as circASAP1 [32], circPPM1F [57] has-circ-0005567 [58]. However, whether circRNAs mediate TAM in PCa has not been elucidated. Our data show that circSMARCC1 is involved in TAM infiltration and polarization through the CCL20-CCR6 signaling pathway. The evidence is as follows: First, PCa and mouse xenograft tissues with high expression of circSMARCC1 exhibited increased numbers of M2 macrophages (CD68^+^, CD163^+^ and CD206^+^) and upregulation of CCL20 expression. Second, conditioned medium (CM) from PCa cells overexpressing circSMARCC1 or the addition of CCL20 recombinant protein alone promoted the recruitment and polarization of M2 macrophages, which could be reversed by CCR6 neutralizing antibodies. Overall, these results reveal a critical role for circSMARCC1 in mediating macrophage infiltration and M2 polarization in PCa. In cancers with M2 macrophage-mediated TME, the CCL20-CCR6 axis may be a promising therapeutic target.

## Conclusions

**Fig. 8:**
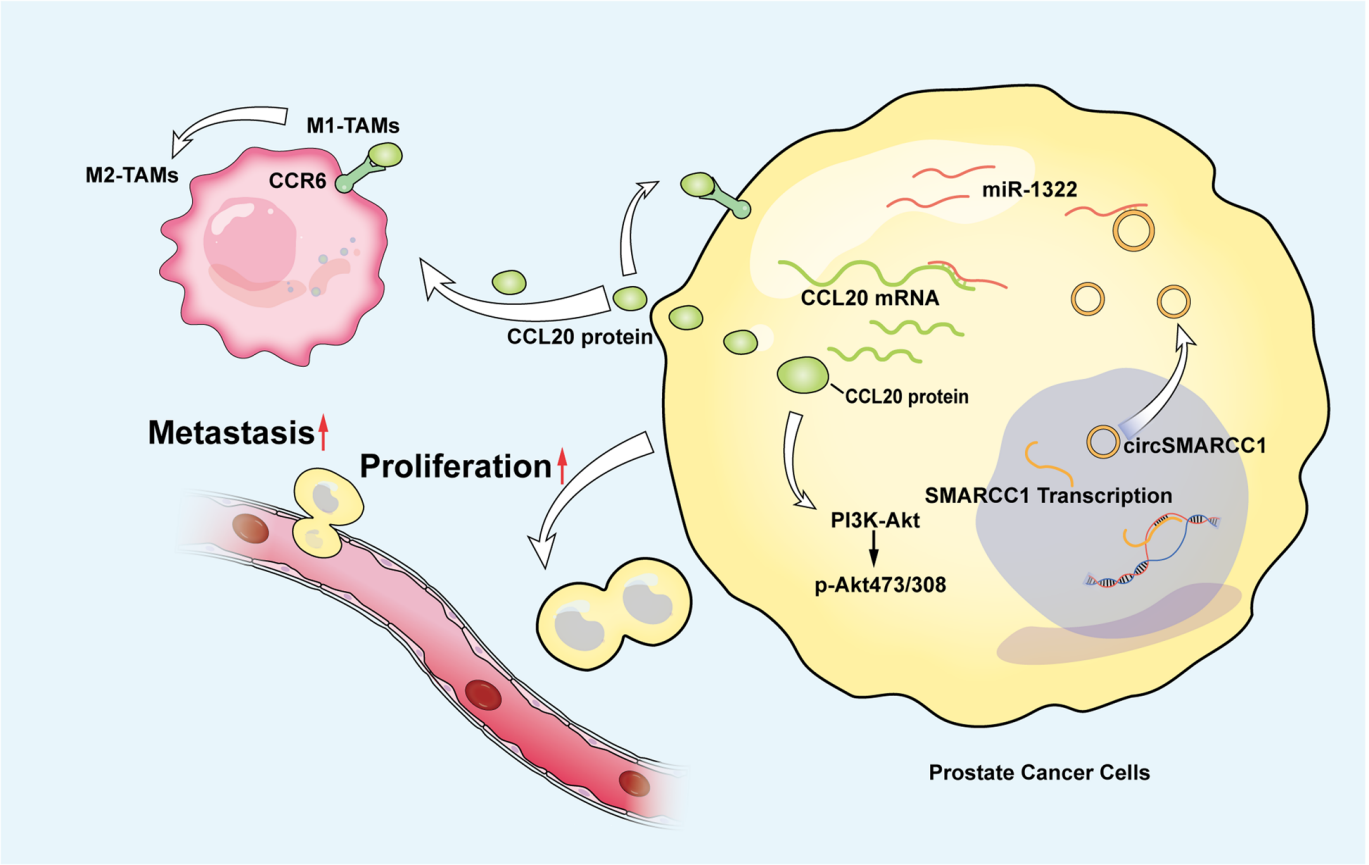
A schematic illustration of the molecular mechanism of circSMARCC1 in promoting PCa progression

Collectively, we identified circSMARCC1 as an oncogenic regulator in PCa growth and metastasis by sponging miR-1322/CCL20 axis and activating the PI3K/AKT signaling pathway. Furthermore, circSMARCC1 disrupts the crosstalk between TAMs and PCa cells via the CCL20-CCR6 axis, including recruitment of TAMs and mediating M2 macrophage polarization, thereby facilitating the progression of PCa (Fig. 8).

### Supplementary Information

**Additional file 1.**

## Data Availability

The datasets used and/or analyzed during the current study are available within the manuscript and its supplementary information files.

## Abbreviations

CircRNA: Circular RNAs
PCa: Prostate cancer
mPCa: Metastatic prostate cancer
qRT-PCR: Quantitative real-time polymerase chain reaction
ceRNA: Competing endogenous RNA
EMT: Epithelial mesenchymal transformation
IF: Immunofluorescence
FISH: Fluorescence in situ hybridization
H&E: Hematoxylin and eosin
IHC: Immunohistochemistry
ELISA: Enzyme-linked immunosorbent assay
CM: Conditioned medium
CCL20: CC-chemokine ligand 20
TAM: Tumor-associated macrophages
TME: Tumor microenvironment
