# Correction: CircSMARCC1 facilitates tumor progression by disrupting the crosstalk between prostate cancer cells and tumor-associated macrophages via miR-1322/CCL20/CCR6 signaling

**DOI:** 10.1186/s12943-023-01881-0

**Published:** 2023-10-23

**Authors:** Tao Xie, Du-jiang Fu, Zhi-min Li, Dao-jun Lv, Xian-Lu Song, Yu-zhong Yu, Chong Wang, Kang-jin Li, Baoqian Zhai, Jiacheng Wu, Ning-Han Feng, Shan-Chao Zhao

**Affiliations:** 1 Department of Urology, Nanfang Hospital, Southern Medical University, Guangzhou, 510515 China; 2 Department of Urology, the Third Affiliated Hospital of Southern Medical University, Guangzhou, 510500 China; 3 Department of Urology, the Third Affiliated Hospital of Guangzhou Medical University, Guangzhou, 510150 China; 4 Department of Radiotherapy, Affiliated Cancer Hospital & Institute of Guangzhou Medical University, Guangzhou, 510095 China; 5 Department of Radiotherapy Oncology, Yancheng City No.1 People’s Hospital, Yancheng, 224005 China; 6 The Fourth Affiliated Hospital of Nantong University, Yancheng, 224005 China; 7 Department of Urology, Affiliated Tumor Hospital of Nantong University & Nantong Tumor Hospital, No. 30 Tongyang Bei Road, Tongzhou District, Nantong, 226361 China; 8 Department of Urology, Affiliated Wuxi No. 2 Hospital, Nanjing Medical University, Wuxi, 214002 China

**Correction: Mol Cancer 21, 173 (2022)**

**https://doi.org/10.1186/s12943-022-01630-9**

Following publication of the original article [1], the authors requested to update the figures as stated below.

- We request to replace the misused image in C4-2 cells (sh-circ group) in Fig. 3H with the correct image which produced in May 26, 2021 when we conducted the experiment.
- We request to replace the misused images in C4-2 cells in Fig. 4J with the correct images which produced in June 2, 2021 when we conducted the experiment.
- We request to replace the misused image in the C4-2-sh-nc-CM group in Fig. 7F with the correct image which produced in April 14, 2021 when we conducted the experiment.
- We request to replace the misused image in the THP-1-Mø-CM group in Fig. 7H with the correct image which produced in June 3, 2021 when we conducted the experiment.

**Fig. 3:**
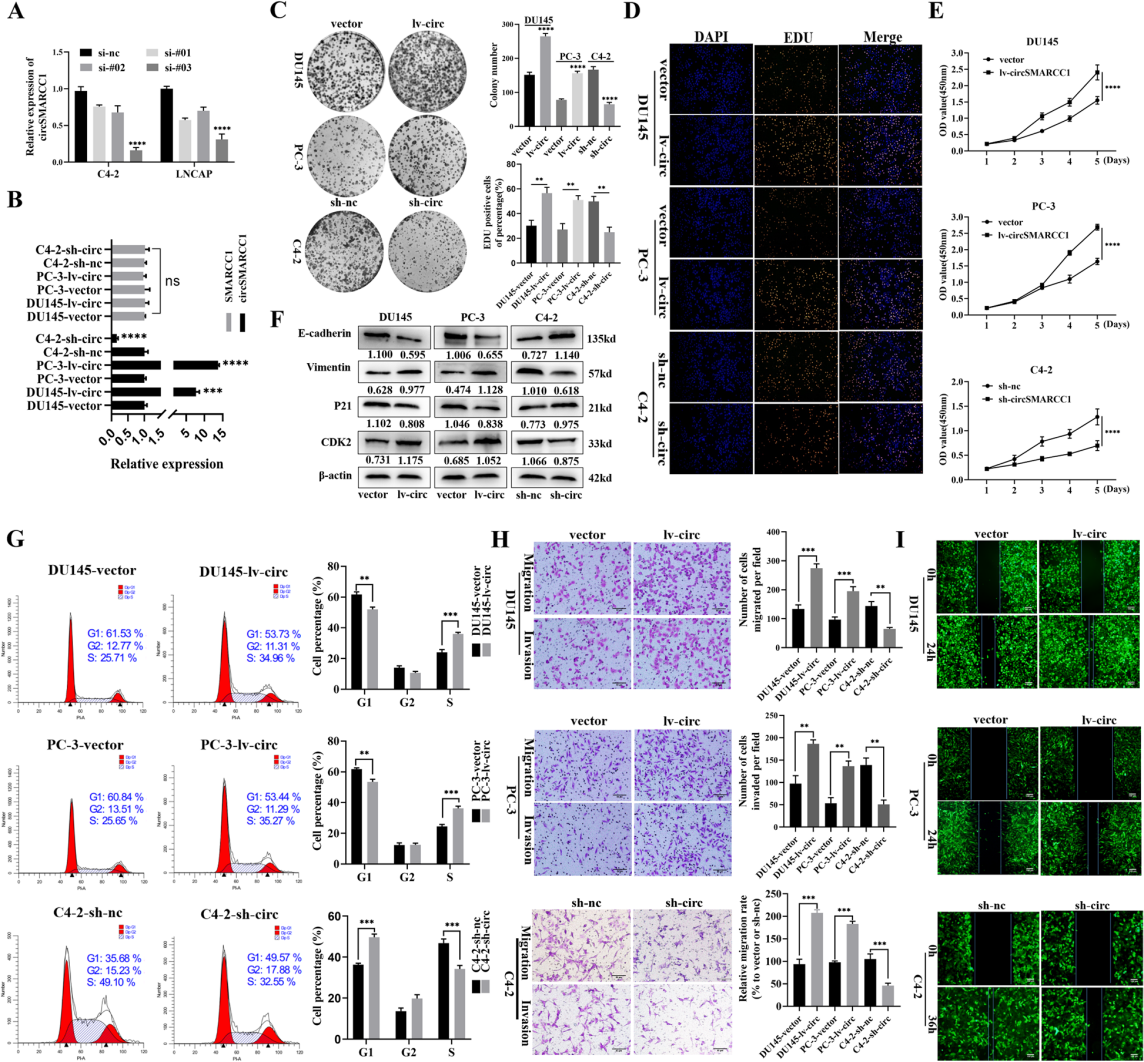
CircSMARCC1 promotes proliferation, migration and invasion of PCa cells. **A** three siRNAs (si-#01,02,03) targeting circSMARCC1 were constructed to silence its expression and confirmed by qRT-PCR. **B** The relative expression of circSMARCC1 and SMARCC1 in PCa cells transfected with circSMARCC1 overexpressing or knockdown lentivirus by qRT-PCR. **C**-**E** Assessment of cell proliferation capacity by colony formation, EdU assay (scale bar, 100 μm) and CCK-8 assay. **F** Western blot analysis evaluated expression of cell cycle-associated proteins and EMT biomarkers following overexpression or knockdown of circSMARCC1. **G** Flow cytometric analysis of changes in the cell cycle profile of PCa cells stably transfected with circSMARCC1. **H**, **I** Transwell assays and wound healing assays assessed the migration and invasion abilities of PCa cells stably transfected with circSMARCC1 (Scalebar, 50 μm). The data are presented as the mean ± SD, **p < 0.01, ***p < 0.001, ****p < 0.0001

**Fig. 4:**
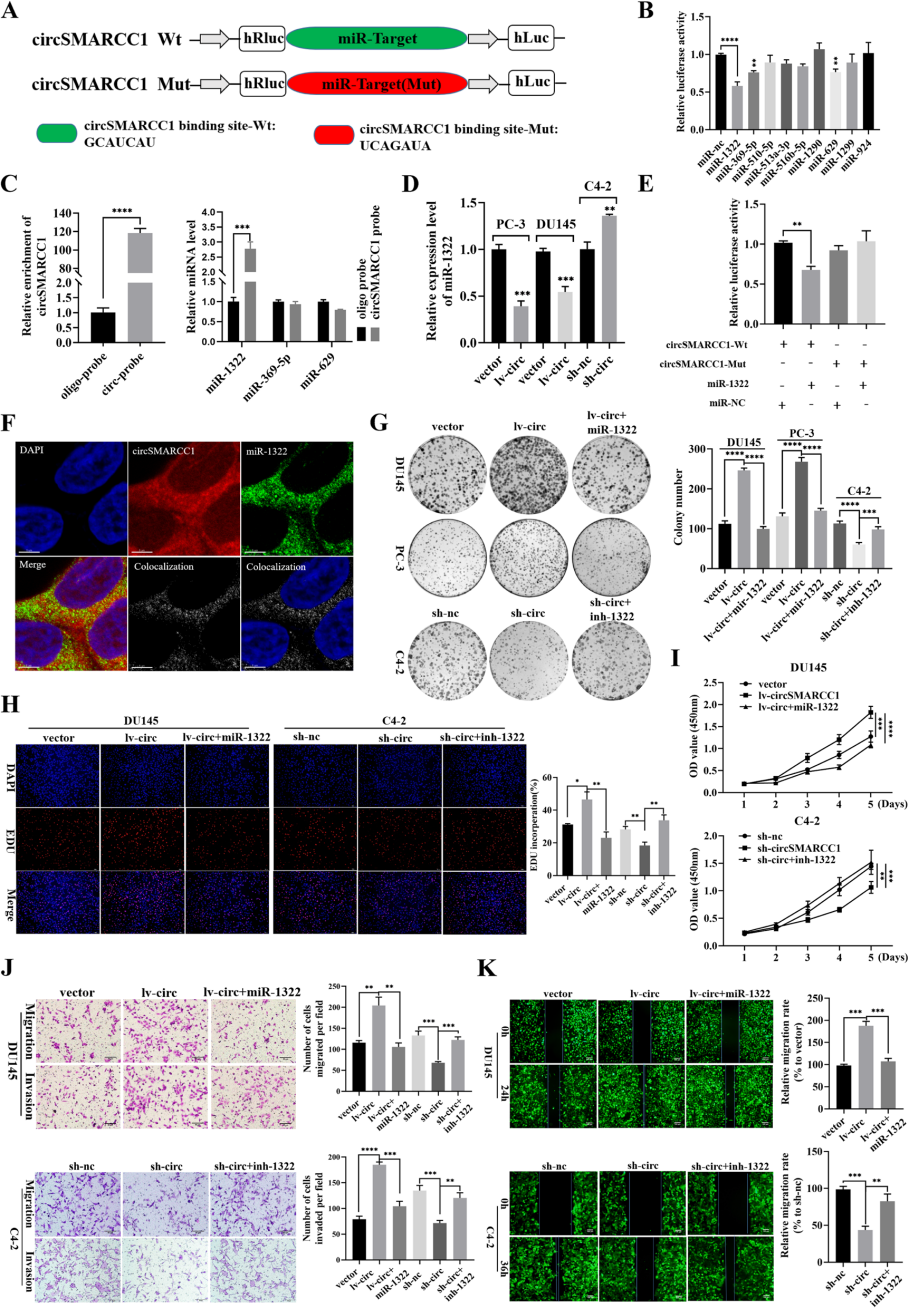
CircSMARCC1 acts as a sponge for miR-1322 and miR-1322 reverses the oncogenic effects of circSMARCC1 on proliferation, invasion and migration in PCa cells. **A** Schematic diagram of circSMARCC1 luciferase reporter vectors carrying wild-type (Wt) or mutant (Mut) miR-1322 binding sites. **B** Luciferase reporter assay to analyze the effects of 9 candidate miRNAs on the luciferase activity of circSMARCC1. **C** The RNA pull-down assay performed in DU145 cells using circSMARCC1 and negative control probes. **D** The relative expression of miR-1322 in PCa cells after transfection of circSMARCC1 was detected by qRT-PCR. **E** The relative luciferase activities measured in 293 T cells co-transfected with circSMARCC1-Wt or circSMARCC1-Mut and miR-1322 mimics or miR-nc by luciferase reporter assay. **F** The co-localization of circSMARCC1 and miR-1322 observed using RNA-FISH in DU145 cells (scale bar, 5 μm). The nuclei were stained with DAPI. **G-I** The viability of PCa cells in miR-1322 rescue experiments was analyzed by colony formation assay, EdU assay (scale bar, 100 μm) and CCK8 assay, respectively. **J**, **K** The migration and invasion capacity of PCa cells in the miR-1322 rescue experiment was analyzed by transwell assays (scale bar, 50 μm) and wound healing (scale bar, 100 μm) assays. The data are presented as the mean ± SD, *p < 0.05, **p < 0.01, ***p < 0.001, ****p < 0.0001

**Fig. 7:**
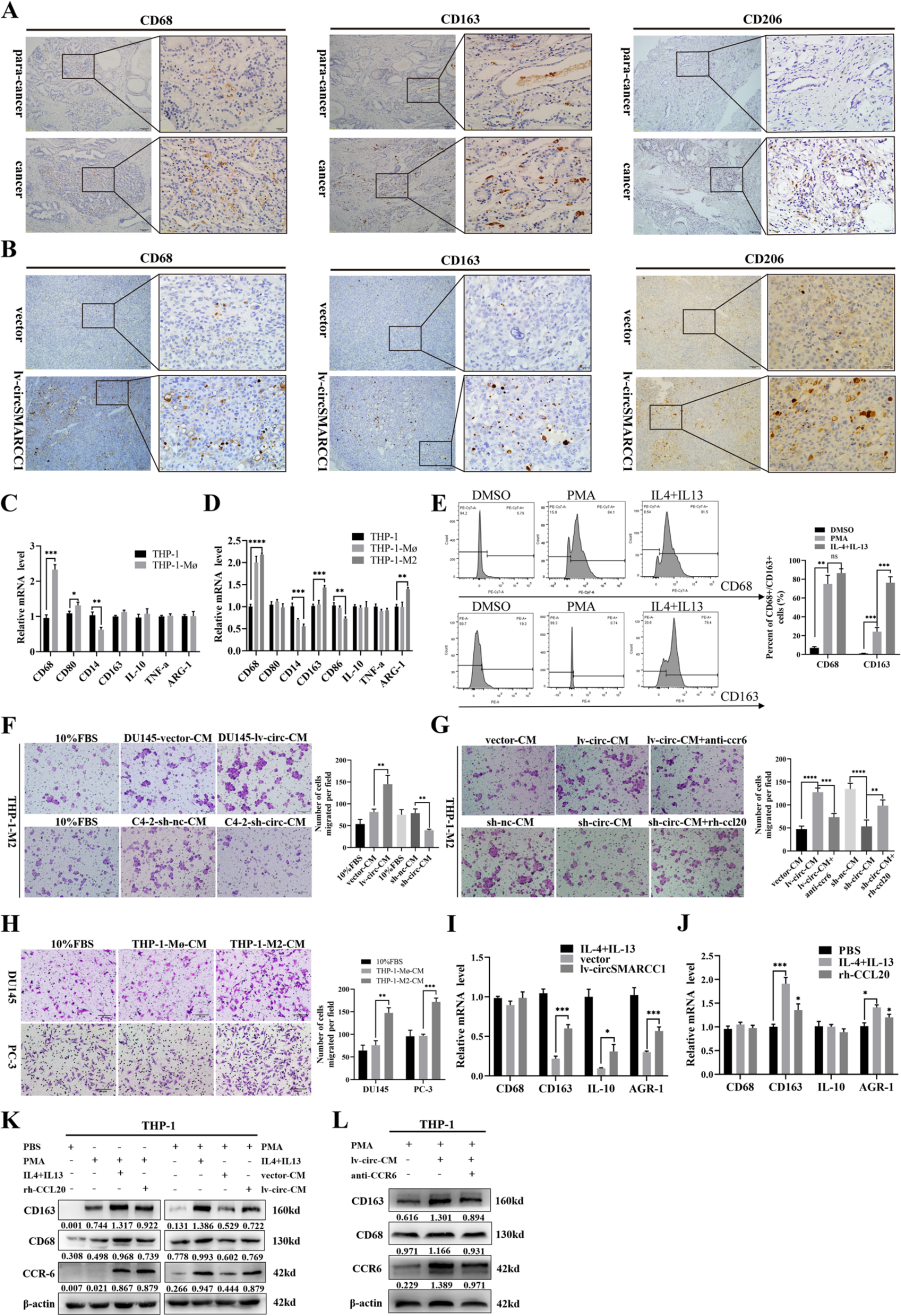
circSMARCC1 promotes M2 macrophage polarization and recruitment via the CCL20-CCR6 axis. **A** IHC detected the infiltration of CD68^+^/ CD163^+^/ CD206^+^ macrophages in human PCa tissue and Para-cancerous tissues (scale bar, 100 μm and 20 μm). **B** IHC detected the infiltration of CD68^+^/ CD163^+^/ CD206^+^ macrophages in mouse xenograft tumors (scale bar, 100 μm and 20 μm). **C**, **D** The expression of M1 phenotype (TNF-a, CD80 and CD86) and M2 phenotype (IL-10, ARG-1 and CD163) markers were detected by qRT-PCR in THP-1 cells, THP-1-Mø and THP-1-M2 cells. **E** The proportion of CD68 and CD163 positive cells detected by flow cytometry. **F** The CM prepared from lv-circSMARCC1 or vector and sh-circSMARCC1 or sh-nc tumor cells was used to evaluate the migration ability of macrophages through transwell experiments (scale bar, 50 μm). **G** The migration ability of TAM was detected by transwell assay using CCL20 recombinant protein and CCR6 neutralizing antibody (scale bar, 50 μm). **H** The CM prepared from THP-1-Mø or THP-1-M2 cells for PCa cells migration experiments (scale bar, 50 μm). **I**, **J** qRT-PCR tested the expression of M2 phenotypes marker (IL-10, ARG-1, CD68 and CD163) when co-cultured with DU145-lv-circSMARCC1 cells or vector cells or using CCL20 recombinant protein, with IL-4 and IL-3 polarization as the reference. **K** The expression of CD68, CD163 and CCR6 in THP-1 cells, THP-1-Mø cells stimulated by CCL20 recombinant protein, and THP-1-Mø cells (with and without co-culture) were detected by Western blotting. **L** The expression of CD68, CD163 and CCR6 in THP-1-Mø cells and THP-1-M2 cells (with and without anti-CCR6) were detected by Western blotting. *p < 0.05, **p < 0.01, ***p < 0.001, ****p < 0.0001.
