# Supplementary material for: CircSMARCC1 facilitates tumor progression by disrupting the crosstalk between prostate cancer cells and tumor-associated macrophages via miR-1322/CCL20/CCR6 signaling
Source: Mol Cancer. 2022 Sep 1;21:173. doi: 10.1186/s12943-022-01630-9 (PMC9434883; doi:10.1186/s12943-022-01630-9)
Supplement: Supplementary file 1 — Additional file 1. [file 12943_2022_1630_MOESM1_ESM.zip › 5.Supplementary_Material.docx]

Supplementary Material

# 1 Supplementary Figures and Tables

# 1.1 Supplementary Figures

**Supplementary Figure 1.** **A** The cluster heat map revealed a more than 1.5 fold change in differentially expressed circRNAs. **B** The scatter plots demonstrated 98 up-regulated and 40 down-regulated circRNAs. **C** The relative expression levels of 5 circRNAs (circMAPKBP1, circRAN, circAHI1, circRBM4 and circSMARCC1) were detected by RT-PCR in 39 pairs plasma samples of PCa and BPH. **D** The melting curve of GAPDH and circSMARCC1 amplified product using the divergent primers. **E** The protein coding potential of circSMARCC1. **F** Assessing the prognosis of circSMARCC1 using Kaplan–Meier survival analysis.

**Supplementary Figure 2.** **A** The relative expression of circSMARCC1 detected by qRT-PCR after the transfection of miR-1322 mimic or inhibitor. **B** qRT-PCR to detect the relative expression of miR-1322 after transfection with miR-1322 mimics or inhibitors. **C** The expression of CCL20 protein and mRNA level was detected by Western blotting and qRT-PCR in the normal prostate epithelial cell line (RWPE-1) and five PCa cell lines. **D** The knockdown efficiency of CCL20 was examined by qRT-PCR analysis. **E** The proliferation ability of PCa cells (DU145 and C4-2) with CCL20 knockdown were detected by CCK8 assay. **F** The migration ability of PCa cells (DU145 and C4-2) with CCL20 knockdown were examined by transwell assay. **G** Western blot analysis of changes in the PI3K-Akt pathway following knockdown of CCL20. **H-I** The proliferation ability of DU145 cells utilizing LY294002 in the rescue experiment was detected by CCK8 assay and colony formation assay. **J** Transwell assay evaluated the migratory capacity of DU145 cells using LY294002 in the rescue experiment. **K** Western blot analysis evaluated expression of cell cycle-associated proteins and EMT biomarkers using LY294002 in rescue experiments. **L** Fluorescence image of metastatic transplanted tumor. **M** The expression of CD31 in xenograft tumors detected by IHC (Scale bar, 100µm and 20µm). **p* < 0.05, ***p* < 0.01, ****p* < 0.001, *****p* < 0.0001.

**Supplementary Figure 3. A** Gene ontology (GO) analyzed the differential function of CCL20 and CCL20 co-expressed genes in PCa. **B** The correlation of CCL20 and CCR6 with macrophages in PCa was analyzed using TISIDB database. **C** The correlation between CCR6 and M2-type macrophages in PCa was analyzed using the TIMER database. **D** TCGA database analysis of the correlation between CCL20 and CCR6 in PCa. **E** The CCK8 assay detected the changes in the proliferation ability of PCa cells (DU145 and C4-2) treated with THP-1-Mø-CM or THP-1-M2-CM. CM, conditioned medium; ns, not significant.

# 1.2 Supplementary Tables

| Supplementary Table 1. Sequences of siRNAs and shRNAs used in this study | |
| --- | --- |
| **siRNA sequences (5’-3’)** | |
| si-h-hsa_circ_0001296_001 | GTGCTGTGATGAGGAAGAT |
| si-h-hsa_circ_0001296_002 | CTGTGATGAGGAAGATGAA |
| si-h-hsa_circ_0001296_003 | TGTGCTGTGATGAGGAAGA |
| genOFFTM st-h-CCL20_001 | ACCGTATTCTTCATCCTAA |
| genOFFTM st-h-CCL20_002 | GACTGCTGTCTTGGATACA |
| genOFFTM st-h-CCL20_003 | GCCAATGAAGGCTGTGACA |

| Supplementary Table 2. miRNA primer sequences used in qRT-PCR and PCR analysis | | | |
| --- | --- | --- | --- |
| **miRNA name** | **types** | **Forward Primer (5’-3’)** | **Reverse Primer (5’-3’)** |
| hsa-miR-1322 | mimic | GAUGAUGCUGCUGAUGCUG | CAGCAUCAGCAGCAUCAUC |
|  | inhibitor | CAGCAUCAGCAGCAUCAUC | - |
| hsa-miR-1299 | mimic | UGAUUGGUACGUCUGUGGGUAG | CUACCCACAGACGUACCAAUCA |
| hsa-miR-510-5p | mimic | UACUCAGGAGAGUGGCAAUCAC | GUGAUUGCCACUCUCCUGAGUA |
| hsa-miR-1290 | mimic | UUACAGUUGUUCAACCAGUUACU | AGUAACUGGUUGAACAACUGUAA |
| hsa-miR-516b-5p | mimic | AUCUGGAGGUAAGAAGCACUUU | AAAGUGCUUCUUACCUCCAGAU |
| hsa-miR-513a-3p | mimic | UAAAUUUCACCUUUCUGAGAAGG | CCUUCUCAGAAAGGUGAAAUUUA |
| hsa-miR-924 | mimic | AGAGUCUUGUGAUGUCUUGC | GCAAGACAUCACAAGACUCU |
| hsa-miR-369-5p | mimic | AGAUCGACCGUGUUAUAUUCGC | GCGAAUAUAACACGGUCGAUCU |
| hsa-miR-629 | mimic | UUGCUAGUUGCACUCCUCUCUGU | ACAGAGAGGAGUGCAACUAGCAA |

| Supplementary Table 3. Primer sequences used in qRT-PCR and PCR analysis | | |
| --- | --- | --- |
| **mRNA name** | **Forward Primer (5’-3’)** | **Reverse Primer (5’-3’)** |
| circSMARCC1 | TGTGTGTGCTGTGATGAGGAAGATG | CATTATCTTCCCCAAGGTCAACTGA |
| SMARCC1 | AGCTGTTTATCGACGGAAGGA | GCATCCGCATGAACATACTTCTT |
| CCL20 | TGCTGTACCAAGAGTTTGCTC | CGCACACAGACAACTTTTTCTTT |
| GAPDH | CAGTCAGCCGCATCTTCTT | GACAAGCTTCCCGTTCTCAG |
| U6 | CTCGCTTCGGCAGCACA | AACGCTTCACGAATTTGCGT |
| MS4A1 | TGCACCCATCTGTGTGACTG | TTCCTGGAGTTTTTCTCCGTTG |
| CBFB | AGAAGCAAGTTCGAGAACGAG | CCTGAAGCCCGTGTACTTAATCT |
| ARPP21 | CCAGCCCTTTGTGAATCCC | CCCTGGACAGACTGAGTGAC |
| PHF6 | AGAGGCACGAAGCTGATGTG | AGTGGTAGTGGTATGTCCTGTG |
| FTO | ACTTGGCTCCCTTATCTGACC | TGTGCAGTGTGAGAAAGGCTT |
| MED7 | AGAAACGTCAACGGCTTGAAA | TGAGGCAAATCATCAGGCAAA |
| CD40 | ACATACAACCAAACTTCTCCCCG | GCAAAAAGTGCTGACCCAATCA |
| SLC25A15 | CCTGAAGACTTACTCCCAGGT | GCGATGTTGGCGATTAGTGC |
