# Supplementary figures and images for: CircSMARCC1 facilitates tumor progression by disrupting the crosstalk between prostate cancer cells and tumor-associated macrophages via miR-1322/CCL20/CCR6 signaling
Source: Mol Cancer. 2022 Sep 1;21:173. doi: 10.1186/s12943-022-01630-9 (PMC9434883; doi:10.1186/s12943-022-01630-9)

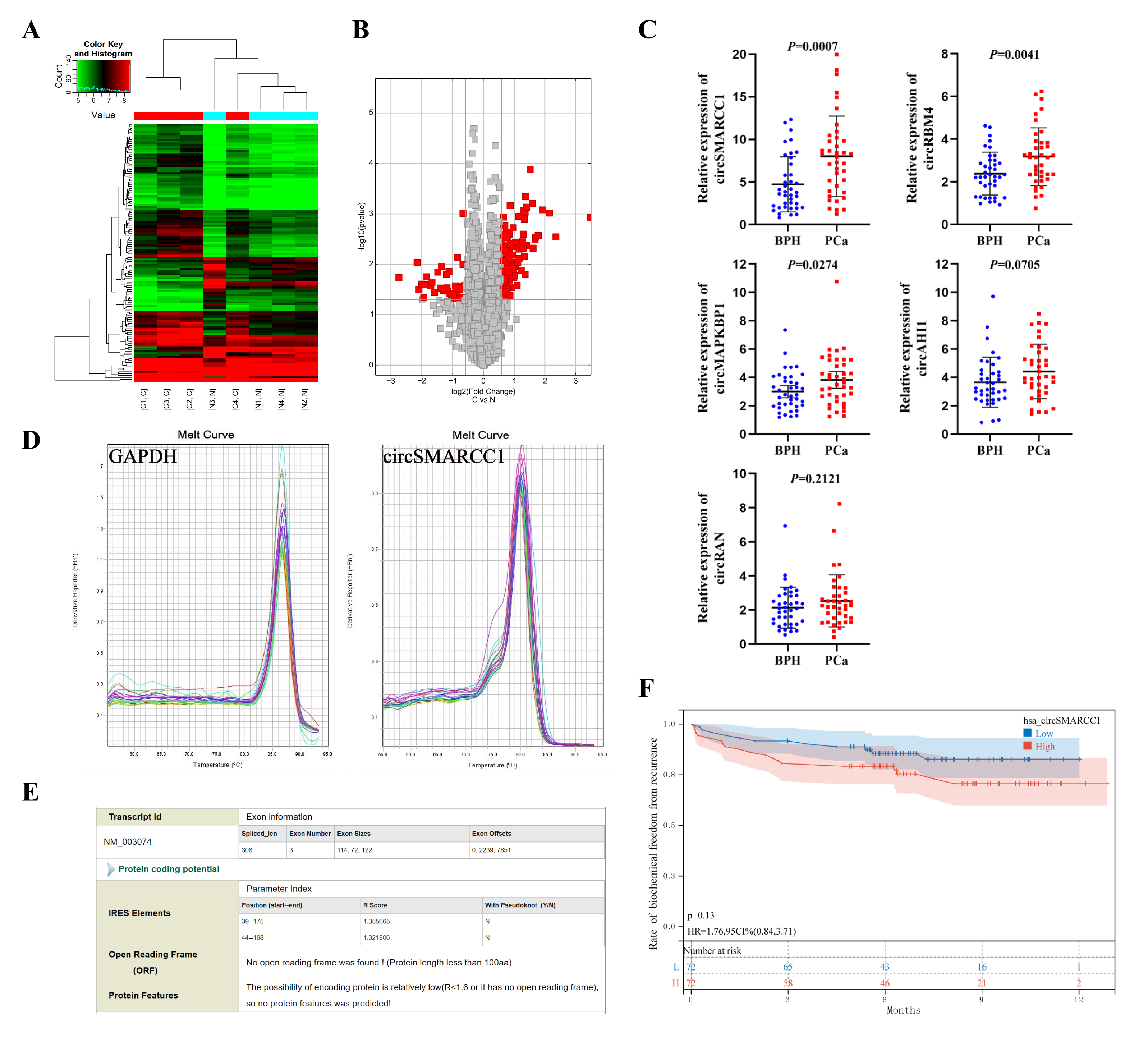

Supplement: Supplementary file 1 — Additional file 1. [file 12943_2022_1630_MOESM1_ESM.zip › Figure. S1.tif]

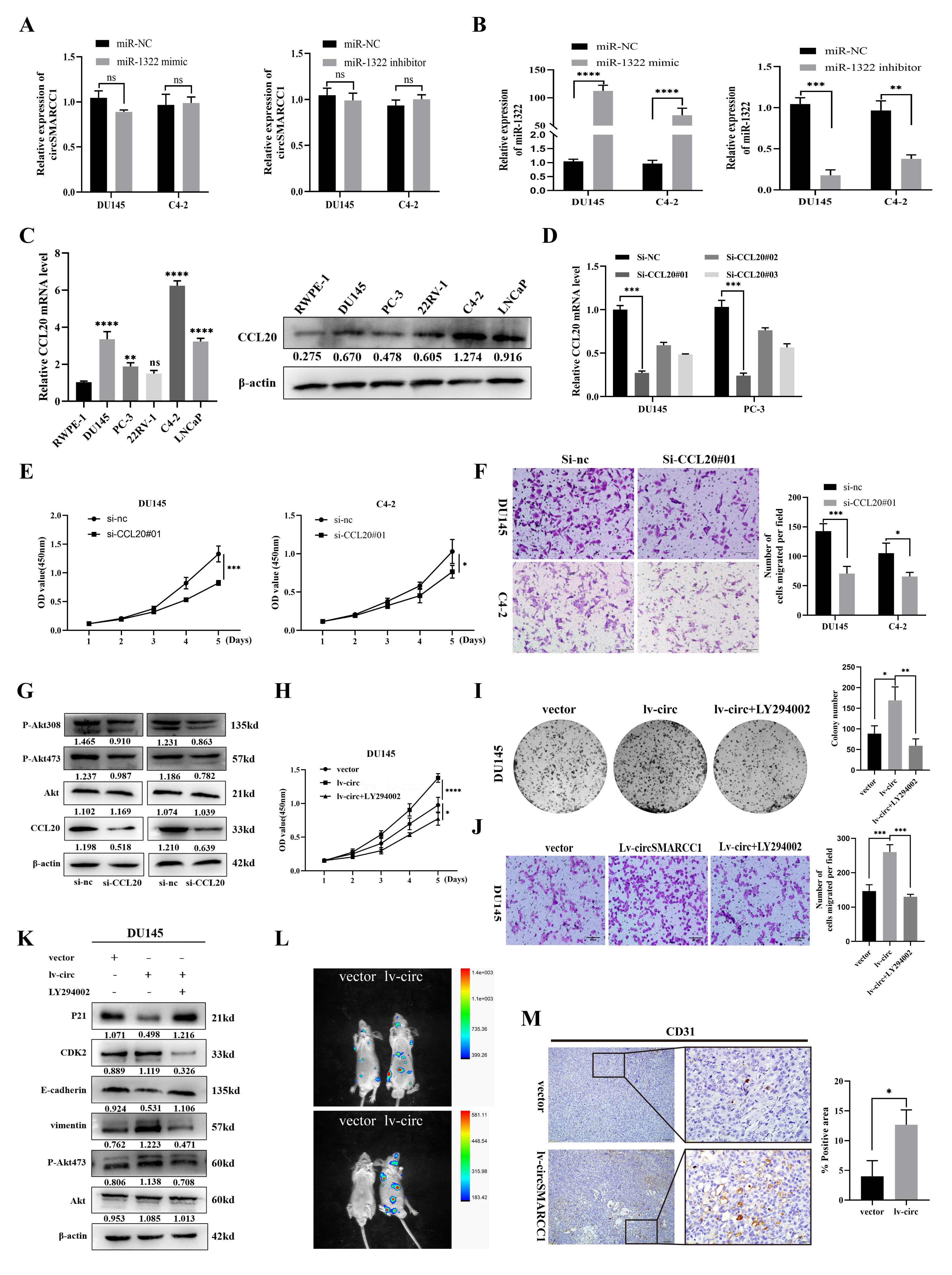

Supplement: Supplementary file 1 — Additional file 1. [file 12943_2022_1630_MOESM1_ESM.zip › Figure. S2.tif]

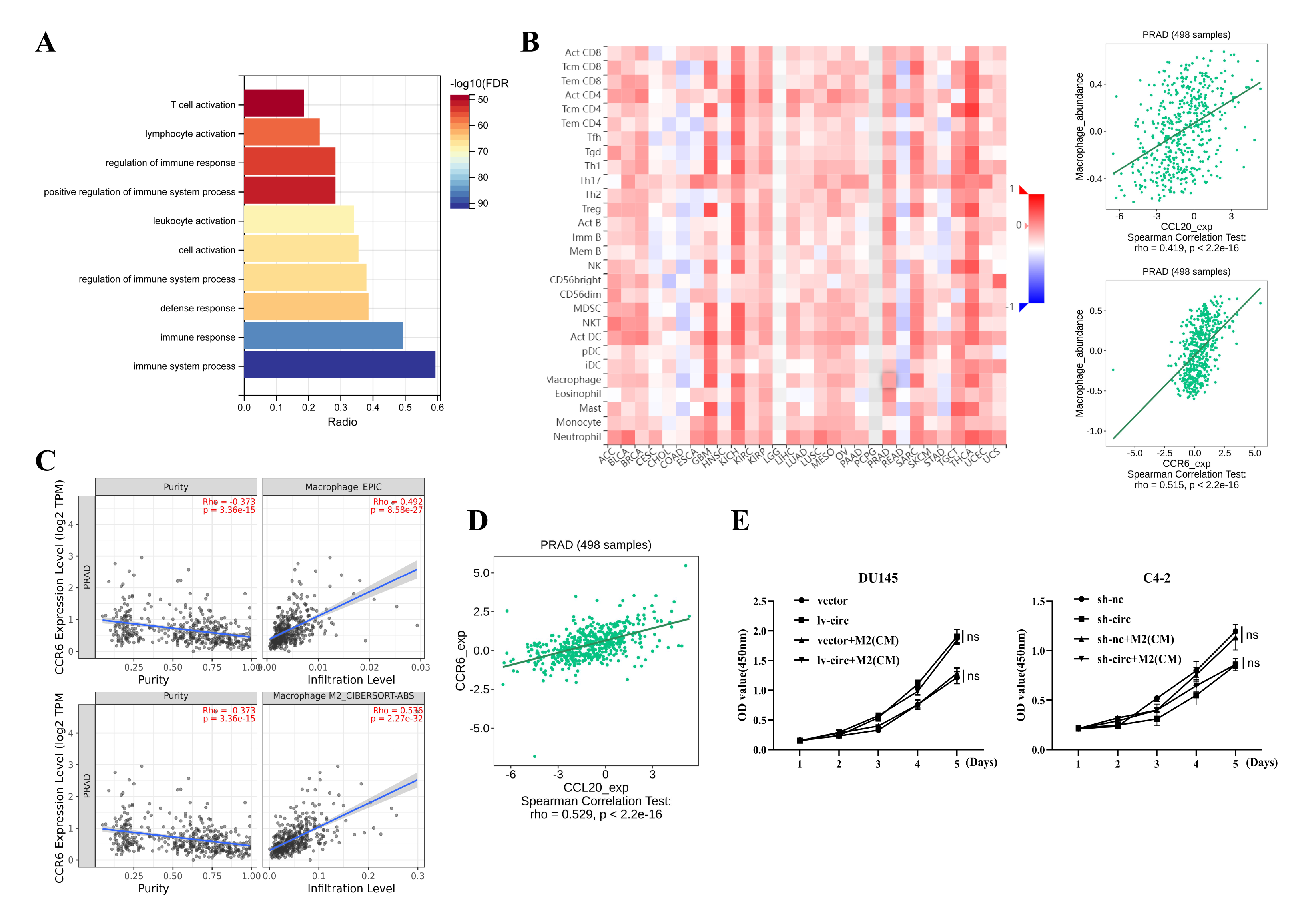

Supplement: Supplementary file 1 — Additional file 1. [file 12943_2022_1630_MOESM1_ESM.zip › Figure. S3.tif]
